# Phi fluctuates with surprisal: An empirical pre-study for the synthesis of the free energy principle and integrated information theory

**DOI:** 10.1371/journal.pcbi.1011346

**Published:** 2023-10-20

**Authors:** Christoffer Lundbak Olesen, Peter Thestrup Waade, Larissa Albantakis, Christoph Mathys

**Affiliations:** 1 Interacting Minds Centre (IMC), Aarhus University, Aarhus, Denmark; 2 Department of Psychiatry, University of Wisconsin, Madison, Wisconsin, United States of America; 3 Tranlational Neuromodeling Unit (TNU), University of Zurich and ETH Zurich, Zurich, Switzerland; University College London Institute of Neurology, UNITED KINGDOM

## Abstract

The Free Energy Principle (FEP) and Integrated Information Theory (IIT) are two ambitious theoretical approaches. The first aims to make a formal framework for describing self-organizing and life-like systems in general, and the second attempts a mathematical theory of conscious experience based on the intrinsic properties of a system. They are each concerned with complementary aspects of the properties of systems, one with life and behavior, the other with meaning and experience, so combining them has potential for scientific value. In this paper, we take a first step towards such a synthesis by expanding on the results of an earlier published evolutionary simulation study, which show a relationship between IIT-measures and fitness in differing complexities of tasks. We relate a basic information theoretic measure from the FEP, surprisal, to this result, finding that the surprisal of simulated agents’ observations is inversely related to the general increase in fitness and integration over evolutionary time. Moreover, surprisal fluctuates together with IIT-based consciousness measures in within-trial time. This suggests that the consciousness measures used in IIT indirectly depend on the relation between the agent and the external world, and that it should therefore be possible to relate them to the theoretical concepts used in the FEP. Lastly, we suggest a future approach for investigating this relationship empirically.

## Author summary

Two influential theoretical frameworks in cognitive science, neuroscience and computational biology, are *the Free Energy Principle* and *Integrated Information Theory*. The first is a formal approach to self-organization and adaptive behavior ‐ in short, life ‐ based on first principles from statistical physics. The second is an attempt at formally describing the intrinsic experience of a given system, that is, how it feels to be that system. In this way, these two theories provide tools for understanding two complementary aspects of a given organism; namely, how it acts in a goal-directed manner based on statistical beliefs about the world, and how it feels to be that system in that process. In this paper, we provide an initial numerical investigation of the potential relation of these theoretical frameworks. We simulate agents that undergo evolution, and show that as their level of integration (Φ, a measure from Integrated Information Theory) increases, information theoretic surprisal (a quantity used in the Free Energy Principle) decreases. We also see that Φ and surprisal fluctuate together, and that these fluctuations depend on sensory input. Finally we provide considerations for future simulation work, and how to bring these two theoretical frameworks closer together.

## Introduction

In recent years, two influential theoretical fields have emerged which provide tools for understanding the relation between self-organization, complexity, beliefs and experience within any given system. These are Integrated Information Theory (IIT) [1,2] and the Free Energy Principle (FEP) [3–5]. They have much in common: both are substrate-agnostic theories that generalize beyond human brains; they take hierarchical and multi-scale perspectives; and they provide formalizations and quantitative measures with origins in information theory (FEP: [6,7], IIT: [8,9]). At the same time, they have potentially complementary outlooks. IIT is grounded in axioms from phenomenal consciousness, the FEP in first principles from statistical physics. IIT is primarily occupied with a system’s internal causal structure and corresponding conceptual structure and intrinsic experience; The FEP has an extrinsic focus on a system’s sensorimotor environmental exchanges and the generative model structure and statistical beliefs they imply. In IIT, existence is an irreducible causal structure [10], while in the FEP it is the presence of a stable Markov Blanket and corresponding (non-equilibrium) steady state [5]. While they are very different, the perspectives of IIT and the FEP are not necessarily mutually exclusive. Indeed, a synthesis of the two theories could provide tools for a formal exploration of the relation between experience and behavior in self-organizing systems in general. We therefore in this paper make an initial empirical exploration of the relation between theoretical constructs from the two theories. We first outline the two theories from their own perspectives, and give some initial theoretical considerations of how the theories could complement or enrich each other. We then present a simulation study that shows a relation between quantitative measures related to each theory, namely Φ and information theoretic surprisal, on both evolutionary and behavioral timescales. Finally, we discuss suggestions for further steps to empirically relate the Free Energy Principle and Integrated Information Theory.

### Integrated Information Theory (IIT)

The fundamental question asked in IIT is which properties a physical system must exhibit in order to support consciousness. It is thus not assumed that consciousness is exclusive to brains. Instead, IIT focuses on the relationship between consciousness and physical systems in general [1,11]. At the heart of IIT lies a distinction between intrinsic and extrinsic descriptions of a system, i.e., what a system is to itself and what it is to an outside observer. The claim is that conscious experience exists exclusively for the system having the experience, so it cannot be modeled as an extrinsic property and must be addressed from the intrinsic perspective of a system. Throughout this paper we use the terms intrinsic and extrinsic in the sense provided by IIT, where a description of a system from its intrinsic perspective may only refer to internal states and excludes, for example, correlations between internal and external states.

One of the novelties of IIT compared to other theories of consciousness is that it takes its outset in the phenomenology of consciousness itself by proposing five axioms that are necessarily true for any conscious experience (in the sense of being logically irrefutable [10]). These axioms are then translated into a corresponding set of postulates about the characteristics that physical systems must have in order to comply with each of the axioms, which in turn forms the basis of the formalism of IIT (for a full technical description of these axioms and postulates in the version used in our work, see [2] (“IIT 3.0”)).

In essence, IIT claims an identity between intrinsic existence and consciousness. The theory builds on the idea that to exist is to exert causal power, i.e., in order for something to be a single coherent thing, it must, as that thing, have the potential to affect something in some way (note that to exert causal power is not necessarily to cause something, but just to have the power to do so). According to this view, the difference between extrinsic and intrinsic existence lies in whether the “thing” exerts causal power upon something else, in which case it exists for the other, or exerts causal power upon itself, in which case it exists for itself. In addition, for something to exist as a coherent whole, it must be irreducible to its parts. This is key to what IIT attempts with its formal analysis. The formalism of IIT can be viewed as an attempt at modeling such intrinsic existence from an observer’s point of view. Since existence is described in causal terms, this formalism then amounts to an analysis of the causal structure of the system itself (not the way the system interacts with its environment). As the analysis specifically needs to represent the intrinsic nature of the system, it is not appropriate to merely use conventional information theoretic measures, as these are inherently extrinsic [1,12]. Instead, IIT defines an intrinsic information measure, loosely described as ‘differences that make a difference’ [2]. It is important to stress that this is very different from, and not directly comparable with, other information measures. Simply put, the intrinsic information of IIT measures how much something, in causal terms, constrains the state of the system it is a part of.

As such, there are two levels of analysis: the mechanism level and the system level. The aim of the mechanism level analysis is to identify all the parts of the system that intrinsically exist in a compositional manner. To exist within the system, the parts must specify irreducible intrinsic information about other parts of the system (such parts are called “concepts”), which is measured by the mechanism integrated information φ. The basic idea behind integrated information φ is to find the minimum difference in intrinsic information between the constraints specified by a mechanism within the system and any possible partition of it. If there is a way to partition it which results in no loss of intrinsic information, the mechanism can be reduced to those parts. However, if there is no such partition, it means that the mechanism specifies information above and beyond its constituents and is therefore irreducible to those constituents. Next, one must evaluate whether the causal structure composed of all these parts is in itself irreducible. All concepts of a system, taken together, form a joint causal structure for the system, called a conceptual structure. At the system level of analysis, this structure is evaluated according to the same principles of integrated information just outlined. The integrated conceptual information Φ quantifies the extent of irreducibility in the conceptual structure.

Note that due to IIT’s fifth axiom and postulate of consciousness (exclusion), the causal structure doesn’t just need to be irreducible, it needs to be the most irreducible out of all overlapping sets and (in principle) across all spatial and temporal grains. Thus, according to IIT a conscious system is one which specifies a causal structure that is *maximally* irreducible. The whole analysis is thus repeated for all possible candidate systems (subsets of elements), while treating the elements outside the candidate system under analysis as background conditions (considering a brain, the sensory input, the rest of the body and the environment are considered background conditions). A system with a non-zero Φ that does not overlap with another set of elements with a greater Φ value (in both time and space) is called a “complex”. In general, the Φ value of a complex is interpreted as the size or the amount of consciousness, whereas the conceptual structure is interpreted as the content or richness of that conscious state, while the individual concepts are the distinguishable things or features constituting this content [1,13].

Note that the computational procedure of calculating Φ is costly, as it is iterating over all possible combinations of system elements at multiple levels, resulting in an explosion of the time required to calculate Φ as systems get larger [14]. This means that it is usually far from feasible to actually calculate Φ on any network much larger than about 10 nodes, far below the roughly 100 billion neurons of the human brain. Thus, it is as yet not possible to relate the findings of any given IIT analysis to the concrete phenomenology that we are familiar with, or to concrete human behavior (but see [13,15]). IIT evaluates causal constraints within the system. In general, the relationship between IIT’s intrinsic measures and the extrinsically observed behavior of even simple systems is yet to be determined, which is a motivation for relating IIT to theories of behavior.

### The Free Energy Principle (FEP)

The Free Energy Principle (FEP) is a theoretical framework, based on first principles from statistical physics and information theory, which attempts to bridge as diverse fields as evolutionary biology, cognitive science and philosophy of mind [5,16] It was originally proposed as a unifying framework for understanding brain function [17,18], but has since been expanded to encompass organisms and self-organizing systems in general [4,5,19]. The FEP builds on the claim that any self-organizing system continually returns to a non-equilibrium steady state, i.e., a small set of non-equilibrium states out of all the states it could possibly inhabit, in order to resist entropic fluctuations and stay in existence in a recognizable form. This can be formalized as solving an optimization problem, minimizing the long-term average of the information theoretic surprisal of the system’s sensory exchanges with the external world, based on an implicit generative model. However, evaluating this surprisal is normally not computationally feasible, so a different, computable quantity is used instead, the variational free energy, which tracks how well the generative model is able to explain sensations, and which upper-bounds the surprisal [3]. Consequently, any living, i.e., self-organizing organism, can be described as if it tracked and minimized the variational free energy of its sensory states relative to a generative model, thus providing a general principle for understanding the behavior of self-organizing systems and life in general [19].

A crucial component of the FEP is the presence of a temporally stable Markov blanket. A Markov blanket is a statistical separation between internal and external states of a system, separated by another set of states that form the boundary between the two. Internal states are not affected by, and do not affect, external states, except when mediated through the blanket states. Blanket states are denoted as sensory states when they affect internal states and are affected by external states, and are denoted as active states when the causal direction is opposite. It is proposed that maintaining a Markov blanket is necessary for keeping structural integrity, that it emerges from simple random dynamical processes, and that maintaining a Markov blanket entails maintaining a non-equilibrium steady state and exhibiting behavior which implicitly minimizes variational free energy [5]. In this process, the dynamics of the system’s internal states come to statistically model those of the external states [20]. Note that, in recent work, a path integral reformulation of the FEP is proposed that does not require assuming a steady state [21]; our starting point here, however, is the classical state-based version of the FEP)

Biological systems can then be construed as a nested hierarchy of Markov blanketed systems, where smaller systems form the components of larger systems. Thus a collective of Markov-blanketed macromolecules may form a cell wall creating a stable Markov Blanket on the level above. This can continue upwards, with cells forming blanketed organs, which in turn form human bodies, social groups, cities, ecologies etc., with every level of the nested hierarchy seemingly acting to minimize the variational free energy of its blanket’s sensory states by existing [6].

The variational free energy of a system’s sensory states is always evaluated relative to a generative model of the external (hidden) states generating predictions about sensory states. One way of minimizing the variational free energy is to (variationally) make state estimates, update parameters and infer model structures, which corresponds to (perceptual) inference, learning and structure learning, respectively. Another way of reducing free energy is to act on the environment in order to produce sensory inputs that are expected by the organism, and therefore preferred. In active inference, a framework based in the FEP where agents choose actions that minimize expected free energy, preferences are implemented as prior expectations for sensory inputs ‐ here called *goal priors* ‐ that are immutable, and must therefore be realized in order to minimize free energy [20]. These goal priors are thought to reflect the kinds of sensations the organism usually encounters, a statistical ‘phenotype’, and are thought to be evolutionarily selected. This provides an interpretation of evolution as Bayesian model selection, where variational free energy is minimized over time by producing organisms that imply more adaptive generative models [7]. The FEP and active inference can thus be used to understand processes at both evolutionary, developmental and mechanistic scales [22,23], as well as how organisms and their niches mutually adapt to, and model, each other [24]. Finally, it is important to point out that work based in the FEP and active inference often take an instrumentalist approach, only claiming that some given systems can be described *as if* it performs active inference [25]. There is, however, an ongoing discussion regarding the degree to which a realist interpretation of the computationalist constructs of the FEP is warranted [26–28], and it is argued that the FEP and active inference are compatible with other justifications for computational and representational realism, like mechanical accounts of computation [29] and teleosemantics [30].

A few different approaches have been taken to apply the FEP to questions of consciousness. Firstly, a variety of active inference Markov Decision Process (MDP) models have been developed, where agents model the world as discrete state transitions and select actions that minimize variational free energy [31]. These can be taken as abstract models that describe the beliefs about the world implicit in a system’s behavior; it is thought, however, that the system’s internal states parameterize these models [32], and they can be related to variational message passing schemes for predictive coding [33]. Because inferences about the world are thought to be the basis for conscious experience, MDP models are thought to reflect the subjective experience of the system that imply them (as for example in [34]). In general, it is argued that conscious experience is a product of inferential rather than sensory processes, and that it emerges when a system’s implied generative model is temporally and counterfactually deep, that is, includes inferences about what will happen in the future and what could have happened in the past given different actions [35,36]. Additionally, it has been argued that consciousness is tentatively linked to the system’s model being responsive to interoceptive information [37], and to the fact that complex agents come to attribute high certainty to mid-level predictions while allowing higher-level beliefs to vary, thus creating a chasm between the world itself and immediate experiences of it [38]. It has also been argued that consciousness is related to affect, and that affect is related to moving towards a variational free energy minimum, suggesting that consciousness arises when explicit evaluation of the expected variational free energy associated with action policies is required, as opposed to when behavior is automatic or reflexive [39,40]. An active inference version of the global neuronal workspace theory of consciousness has been formulated [41], and an active inference neurocomputational Projective Consciousness Model, also based axiomatically on phenomenology, has been proposed to explain the perspectival and integrated quality of first person experience [42,43]. There has also been an attempt at employing generative modeling techniques on phenomenological experience, linking ‘raw’ immediate experience to an active inference agent’s observations, and the total experience to the best explanation or most likely model given those experiences [44]. A more detailed discussion of how the mathematical constructs of the FEP potentially relates to consciousness can be found in [32], with the essential point being that the mind-matter duality can be grounded in the difference between the intrinsic information geometry of the probabilistic evolution of internal states, and the extrinsic information geometry of probabilistic beliefs about external states that is parameterized by the internal states (note that ‘intrinsic’ and ‘extrinsic’ are here used in a different sense than the IIT-based one we use). These accounts, even as they differ substantially, are all based in predictive processing accounts of brain function, which is argued to be a fruitful framework within which to identify neural correlates of consciousness [45].

We do not in this paper go in depth with the theoretical relationship between IIT and the FEP, but instead focus on a numerical investigation of some core constructs from the two theories. We will first, however, take a moment to outline some considerations as to the potential for mutual enrichment between Integrated Information Theory and the Free Energy Principle.

### Motivation for relating the FEP and IIT

There are multiple ways in which IIT and the FEP appear to be compatible, and where there seems to be potential for mutual contribution. In a brief discussion, [32] argue that there is a construct validity between the two approaches, claiming that the five axioms of IIT all are compatible with Markov Blanketed variational free energy minimization systems as conceptualized under the FEP. It is speculated that relating the FEP to IIT might help distinguish between conscious and unconscious types of active inference processes, and possibly be a step towards a unitary concept of consciousness. They also point out that both theories rest upon partitions of causally related systems, which, in addition to further indicating that the theories might be mutually intelligible, also highlights possible avenues for research. See Albantakis (2020) for a brief discussion and criticism of these claims.

The FEP and IIT take different, but potentially compatible, ontological starting points. IIT is physicalist, in the sense that it is occupied with describing the causal relations between physical states within a system, and it is intrinsic, in the sense that it exclusively considers causal relations within the system, and only within a single point in time (at a particular temporal grain). FEP is functionalist, in that it considers abstract computational beliefs and their expression in behavior, and often extrinsic, in that it is interested in the relation between the system and its surrounding environment. Better understanding the relationship between such intrinsic/physicalist and extrinsic/functionalist accounts could provide value both for IIT and the FEP. To IIT it might provide a means for making inferences about a system’s intrinsic perspective based on an extrinsic account of situated behavior (which often is the only account accessible for real biological systems). For the FEP it could provide a way to relate the behavior and implied computations in a system (understood instrumentally or not) to its intrinsic causal structure and level of integration.

The two different conceptualizations of when something exists as a ‘thing’, in the FEP meaning maintaining a Markov Blanket over time, and in IIT meaning being a maximally integrated cause-effect structure over a background of environmental influences, might be complementary concepts (see also [46]). It could be conjectured that complexes do not usually cross stable Markov Blankets, so that consciousness is usually contained within, as opposed to extending across, the dynamically maintained borders of organisms. Methods from IIT might also complement the FEP by determining at which level of a hierarchy of nested Markov Blankets consciousness is located, namely the level where intrinsic integration is the highest.

Another place where the two theories might contribute to each other is in the relation between conscious experience and the external world. IIT is concerned with the experience of a system regardless of its surroundings (but given its background conditions), but under the FEP, self-organizing systems must imply models of the environment (parametrized by their internal states) in order to persist. This indicates that IIT-based formal descriptions of a system’s intrinsic experience might, to some degree, reflect its external environment. It might even be possible to show that a system’s implied generative model and beliefs are to some degree reflected in its conceptual structure, a kind of integrated representationalist interpretation of the FEP. Additionally, in FEP organisms are often thought to align their implied generative models (and consequently the internal states that parametrize them) through (social) interaction with their conspecifics, which could involve alignment of their internal causal structures, conceptual structures and intrinsic experience.

Finally, IIT is agnostic as to whether or not a conscious system self-organizes (although [46] relate integration to self-maintenance). The FEP states, however, that self-organizing systems maintain their structure and return to a stable subset of states by minimizing variational free energy. One might surmise that minimizing variational free energy might then be related to maintaining a stable, self-similar and spatially bound consciousness across time, that is, perhaps, a sense of self.

It is also suggested by [32] that one might find a relationship between Φ and variational free energy for a given system, so that minimizing variational free energy simultaneously leads to an increase in Φ. Given that [47] suggest that being more integrated might have evolutionary advantages, which should lead to a better ability to minimize variational free energy, such a relationship does seem likely. The relationship might, however, become more complex, given that both implicit statistical beliefs and surprisal, and also the amount of integration resulting from evolution, vary with the complexity of a given task as well as the constraints on the system, rather than only how well the system performs a task [47]. In this paper, we therefore take first steps towards an empirical investigation of the relation between the two constructs. Importantly, we do not yet compare Φ to the variational free energy of the system, but instead compare it to an empirical approximation of the surprisal associated with the system’s sensory states, since this is the quantity that variational free energy minimization ultimately seeks to minimize. We then leave it for future work to properly incorporate further aspects of the FEP, for example by calculating Φ for a system that performs active inference under a known generative model of the world (See Limitations and Further Work).

It is noteworthy that [48] has attempted to synthesize IIT, FEP and Global Neuronal Workspace theory into a new Integrated World Modelling Theory. It is argued that active inference and the Free Energy Principle can be used to bridge the otherwise contesting extrinsic and intrinsic perspectives of the two other theories, and that this synthesis has applications across a number of areas. It is beyond the scope of this paper, however, to contribute to theoretical work on synthesizing the theories. Instead, we focus on investigating the numerical relation between Φ and empirically approximated surprisal, and discuss the types of research this might lead to in the future.

### Evolving animats

**Fig 1:**
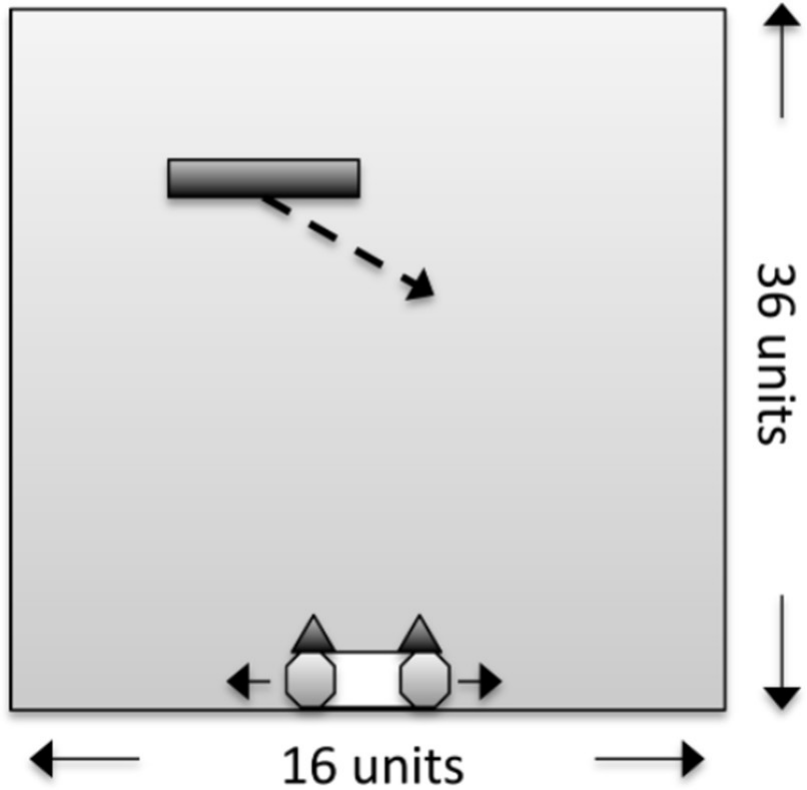
Schematic of the block-catching task in the simulation. A block is falling from top to bottom, either towards the left or the right. The animat has two sensory nodes, which are activated when the block is above them. It also has two motor nodes, which allow it to either move to the right or left. Depending on the size of the block, the animat’s task is to either catch or avoid the block. Reproduced from [47].

To investigate the relationship between the measures of IIT and FEP we replicated the evolutionary simulation presented in [47], where it was found that animats (artificial adaptive agents) on average evolved more concepts and higher values of Φ when the task environment was more complex and difficult, compared to simpler and easier tasks. The animats in the simulation were evolved to perform a simple perceptual categorization task, shown in Fig 1. Within the world, animats inhabited 3 squares of the bottom row of a tetris-like space. On each trial, a block with a certain width would fall from the top to the bottom, eventually either hitting or missing the animat at the end of the trial. Each trial the block would move consistently to the left or right while falling (at one square per time step). The task of the animat was to either catch or avoid the block at the end of the trial depending on the width of the block. This, together with the different falling directions of the block, gave four different task types (right catch, right avoid, left catch and left avoid) and a total of 128 trials (given the maximum of 4 different block sizes). Internally the animat was a Markov brain [49], a network consisting of 2 sensory nodes, 2 motor nodes and 4 hidden nodes. Each sensory node responded on seeing the block directly above it and were placed on the outermost squares of the animat (thus, no sensory input was received from above the middle of the animat). The hidden and motor nodes each had an internal logic determining how different combinations of inputs resulted in either activation or deactivation of the node. The motor notes controlled how the animat moved around in the space, such that when one motor node was active, it would move in a direction corresponding to that node (left or right), and while none or both was active the animat would not move. More details on the animats and the simulation environment are provided in the methods section, and we recommend visiting http://integratedinformationtheory.org/animats.html for a visual presentation of the task and the animat.

In [47], there were 4 tasks, which differed by the sizes of the falling blocks, each with increasing difficulty. Here we only simulated the easiest task (task 1) and the hardest task (task 4). We evolved 150 lines of descent (LOD), 50 for the easy task and 100 for the hard task. Each LOD was evolved over 60000 generations, starting from an unconnected network. For each generation a set of animats would mutate by slightly changing the connections and logics of the hidden nodes, and the best performing animats would form the basis of the next generation. Each LOD represents an independent evolutionary simulation and can for this reason be seen as different species, independently evolved to do the same task. Again, more details are provided in the method section. In addition to comparing the easy task and hard task animats, we also separately analyze the 8 hard task animats with perfect fitness in the last generation. This is done for consistency with [47], and because perfect fitness animats are easier to interpret since they are in fact performing the task correctly. Since more than two thirds of animats reached perfect fitness on the easy task and the overall high fitness achieved on average, we did not separate out perfect fitness animats in the easy task.

Finally, we consider how to extract FEP-related measures for the animat at each timepoint, in order to compare them with Φ. The immediate challenge here is that the variational free energy (and also the surprisal which it upper bounds), in theory, is based on the animat’s implicit generative model of the world, which we do not a priori have access to. In the literature, simulated agents are often constructed with a generative model of their surroundings, based on which they calculate expected free energy given different action policies and select the policy with the lowest expectation (like in [34]). In this case it is possible to track the beliefs and the variational free energy of the agent as it interacts with its environment. The animats are constructed as deterministic logic networks, however, and do not by construction possess a generative model. If one could reconstruct the generative model that the animats must have had in order to generate their observed behavior—that is, their implied generative model—one could directly access their implicit beliefs and free energy on each timestep. It is no simple task to reliably reconstruct such a generative model, however, so we do not do it here (although see Limitations and Further Work for a suggestion on how to do it). Instead of obtaining the variational free energy directly from the generative model, we construct an empirical approximation of the model-conditioned surprisal, the quantity which in the FEP is minimized by minimizing variational free energy. This approximation does not require access to the animat’s implied generative model, and therefore makes possible an initial numerical relation of the IIT and FEP.

Surprisal is calculated as the negative log probability of an observation (i.e., sensory state) occurring, given the animat’s generative model:

ℑ(o∣m)=−ln(P(o∣m))

Where ℑ is the surprisal, and *P(o|m)* is the probability *P* of outcome *o* occuring on the timestep, given the generative model *m*. Calculating *P(o|m)* requires access to the animat’s generative model, and is therefore not accessible here, so we instead approximate it with an *empirical goal prior*. We construct these empirical goal priors by using the distributions of observed sensory states of (perfectly) adaptive animats. The sensory states that perfect animats sample should be the kinds of observations that animats expect, assuming that they *a priori* expect to perform their task well, and have a useful model of their environment. Animats are therefore “surprised” when sampling sensory states that are uncommon under adaptive behavior i.e. different from what the perfect animats encounter. Importantly, we construct different probability distributions for each of the four task types (right catch, right avoid, left catch and left avoid) and at each timestep in a trial, since adaptive animats ought to have different expectations at different task types and timesteps. We additionally calculate surprisal using the sensory state distribution from the perfect animat that results in the lowest surprisal. This means that surprisal is evaluated relative to the kind of perfect behavior that is the most similar to its own, avoiding that rare but still perfect strategies result in high surprisal. The surprisal measure we use here is inherently linked to the fitness of the animats, as should be the case from the perspective of the FEP, but only insofar as the task-optimality of behavior is in fact reflected in an animat’s sensory states within a particular trial. Because it is defined for within-trial time, and because it depends on sensory states rather than the motor states that determine fitness, the model-conditional surprisal is not simply a fitness measure. In addition, surprisal depends on the entropy of the empirical goal prior, so that a deviance from a more uncertain expectation results in less surprise than if it was from a more certain expectation.

Interestingly, using an empirical goal prior to approximate animats’ surprisal allows us to bypass the step of variational free energy entirely. This also means, however, that our measure, even disregarding the ways in which it might be a poor approximation of the model-conditioned surprisal of the FEP, does not directly reflect the epistemic component of the variational free energy (although it might do so implicitly by making animats expect epistemic behavior to the degree that perfect animats exhibit it). Our comparison here should mainly be considered as an initial comparison, until measures based directly on a (reconstructed) generative model can be used.

In the following, we present results from an evolutionary simulation where animats perform the task as described. We replicate some of the findings from Albantakis et al. [47], and additionally show that surprisal decreases over evolutionary time. Moreover, we assess how Φ and surprisal change on within-trial time and how these fluctuations cross-correlate, and relate, in an exploratory manner, these cross-correlations to the general patterns of fluctuations in Φ and surprisal.

## Results

In the following, we first investigate the changes in average Φ, surprisal and fitness on the evolutionary timescale. Next, we consider the within-trial per timestep fluctuations of Φ and surprisal, and how these covary on a non-aggregate level.

Finally, we split the animats into 3 groups based on correlation profiles (negative, positive or no correlation between Φ and surprisal) and investigate how these groups differ in both evolutionary and trial time.

**Fig 2:**
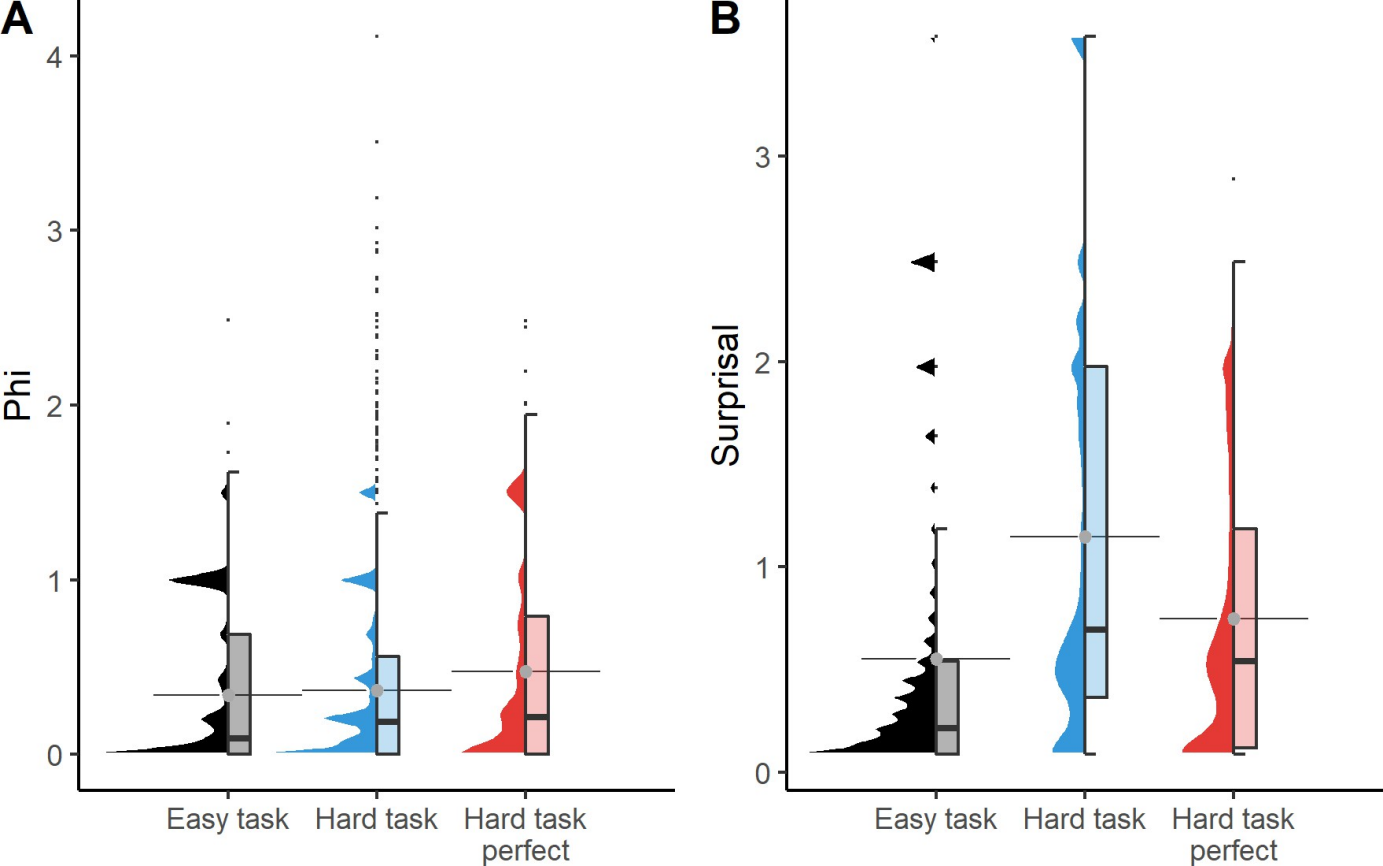
Distributions of Φ and surprisal at the last generation of evolution depicted with half violin plot and half boxplot. Distributions are shown for each task and for the hard task animats that evolved perfect fitness. The gray dots with horizontal lines represent the calculated average of the distributions.

Our data set consists of two task environments (easy and hard) with 50/100 independent evolutions, respectively (Table 1). The distributions of Φ and surprisal at the final generation are shown in Fig 2. It can be seen that the mean and median of Φ is higher in the hard task, and even higher for the perfect animats. In contrast, surprisal is higher on the hard task, but lower for perfect animats. We also see that the mean and median capture differences between the distributions differently. For comparability with Albantakis et. al [47], we subsequently present mean values in the main text (see S1 and S2 and S3 for the same analyses using the median value).

**Table 1:** Descriptive statistics of the data. Note that the number of perfect animats in both tasks are substantially larger than in Albantakis et al. [47]. This is likely due to optimization of the MABE framework, used for the evolutionary simulation, between then and now.

	Easy task	Hard task
Total number of LODs	50	100
Number of LODs with perfect fitness on final generation	34	8
Number of LODs with mean Φ = 0 on final generation	10	6
Number of LODs with at least 1 trial of mean Φ = 0 on final generation	20	31
Percentage of trials with mean Φ = 0 on final generation (out of 128 total trials)	22%	8.3%
Quartiles of Φ values on final generation	min: 0 Q1: 0 Q2: 0.09 Q3: 0.69 max: 2.49	min: 0 Q1: 0 Q2: 0.18 Q3: 0.56 max: 4.11
Quartiles of surprisal values on final generation	min: 0.09 Q1: 0.09 Q2: 0.22 Q3: 0.54 max: 3.58	min: 0.09 Q1: 0.36 Q2: 0.69 Q3: 1.97 max: 3.58

### Changes in evolutionary time

**Fig 3:**
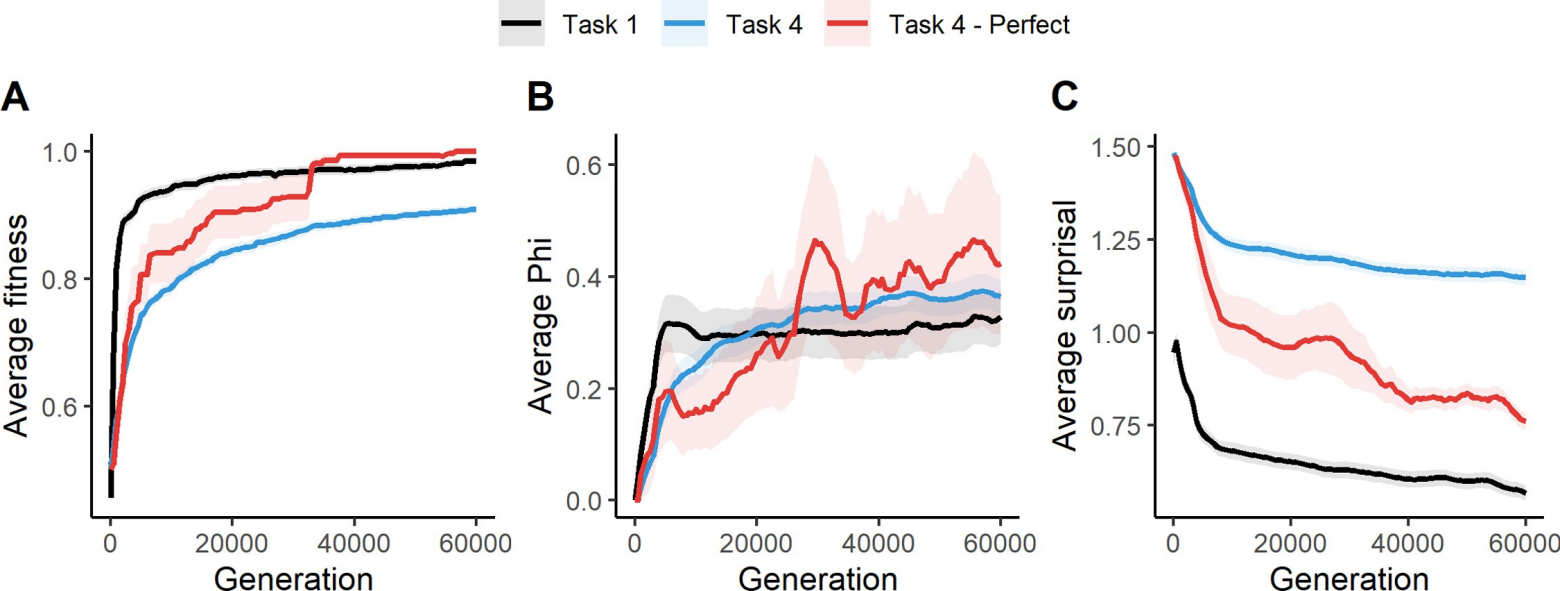
Change in average fitness (A), Φ (B) and surprisal (C) over evolutionary time for the easy task (black), the hard task (blue) and perfect animats in the hard task (red). The variables are first averaged across all values within each animat, then averaged across all LODs at each generation. The ribbons around the lines show the standard error from averaging across LODs.

Fig 3 shows changes in Φ and surprisal over evolutionary time, averaged across LOD’s for each generation. Fig 3A and 3B shows our replication of the main findings in Albantakis et al. [47]. Fitness increases over evolutionary time and is higher in the easy task than the hard task. The red line shows the 8 perfect animats in the hard task, which reach near-perfect behavior about halfway through the simulation. We also see that Φ is higher in the hard task than in the easy task, and slightly higher for perfect animats in the hard task at the end of evolution. Compared to the results of Albantakis et al. [47], we see higher average values of fitness and Φ in all conditions. Additionally, these values increase faster over evolutionary time and are more similar across tasks. This is likely due to a recent increase in the efficiency of MABE’s evolutionary algorithm [50].

Our goal in this study is to compare changes in IIT quantities over the course of the animats evolution to FEP related quantities. As shown in Fig 3C, surprisal decreases over evolutionary time and is lower in the easy task than in the hard task. In the subset of 8 perfect animats in the hard task, surprisal decreases more than on average. However, it never gets as low as in the easy task.

### Fluctuations in trial time

**Fig 4:**
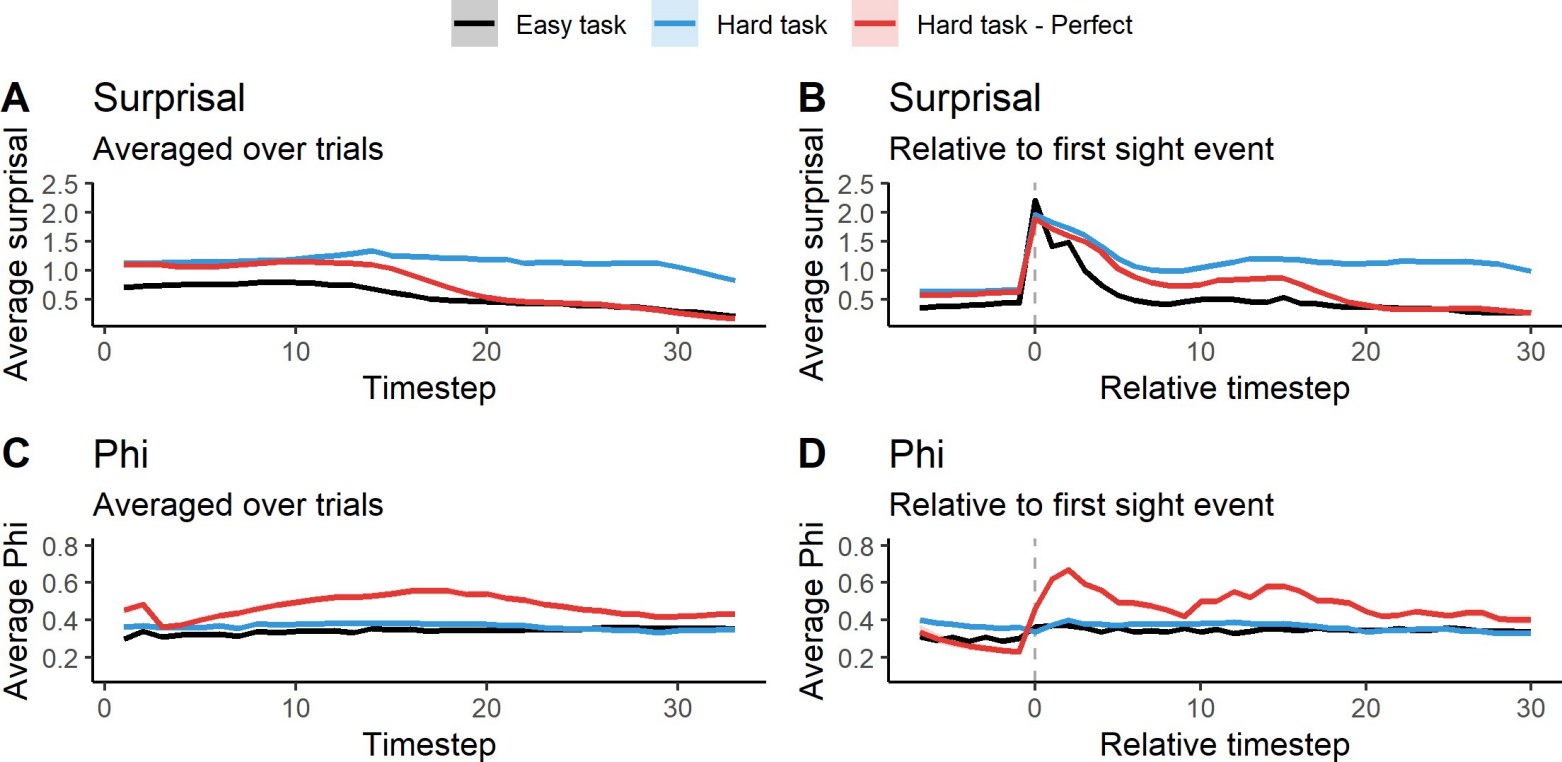
Average Φ and surprisal over trial time for the easy task (black), the hard task (blue) and perfect animats in the hard task (red). A) average surprisal over trial time. B) average surprisal over trial time, centered around the first observation of the block. C) average Φ over trial time. D) average Φ over trial time, centered around the first observation of the block. Shading around lines is the standard error of the mean. See S5 Fig for the variability across LODs. Note that the averages on B and D are based on a decreasing number of trials as the relative timestep gets further away from 0. In order for the relative timestep to go to 30, at a given trial, the animat needs to see the block within the first few real timesteps.

Fig 4 shows Φ and surprisal (averaged over trials and LODs) plotted over trial time (36 time steps) at the end of evolution (generation 60000). Fig 4A shows that surprisal decreases over trial time. The easy task has lower surprisal overall, and decreases further around halfway through the trial. Surprisal is higher in the hard task, but surprisal of the perfect animats decreases rapidly after the middle of the trial, and ends on a similar level as for animats in the easy task. This suggests a shift towards lesser entropy in the goal priors towards the end of the trial (see the methods section for more on this point). Fig 4B shows surprisal when trial time has been centered around the animat’s first observation of the block (marked with a dashed line). Here it can be seen that surprisal increases sharply when the block is observed, but decreases afterwards. Since goal priors are much less entropic at the end of the trial, animats that deviate from the goal prior have higher surprisal than in the early timesteps, leading to another increase for animats in the hard task. The surprisal of the perfect hard task animats decreases after seeing the block, again ending at a level resembling that of animats of the easy task.

In Fig 4C, we see the average Φ over trial time. Here the lines are almost flat for both tasks, with the hard task being very slightly higher at the beginning of the trial, which corresponds to the difference in Φ levels we see at the end of evolutionary time (Fig 3B). For perfect animats there is a general increase in Φ towards the middle of the trial, after which it decreases again. This suggests that the relatively higher average Φ of the perfect animats can be contributed to something that happens during the middle of the trial, as opposed to being a general increase in the level of Φ for this type of animat. Fig 4D shows the average Φ across trials, with trial time centered around the animat’s first observation of the block. At the time of sensing the block, Φ increases slightly for animats in the easy task, and strongly for perfect animats in the hard task, while there seems to be no average difference when including all animats in the hard task. This suggests that the increase in Φ we see for the perfect animats in the Fig 4C is related to the animat’s observation of the block. Note that even though there are differences in average Φ and surprisal, with very small standard errors on the mean due to the large number of simulated trials, there is still large variation between individual animats (see S5 and S6 and S7 Figs).

### Correlations between Φ and surprisal

**Fig 5:**
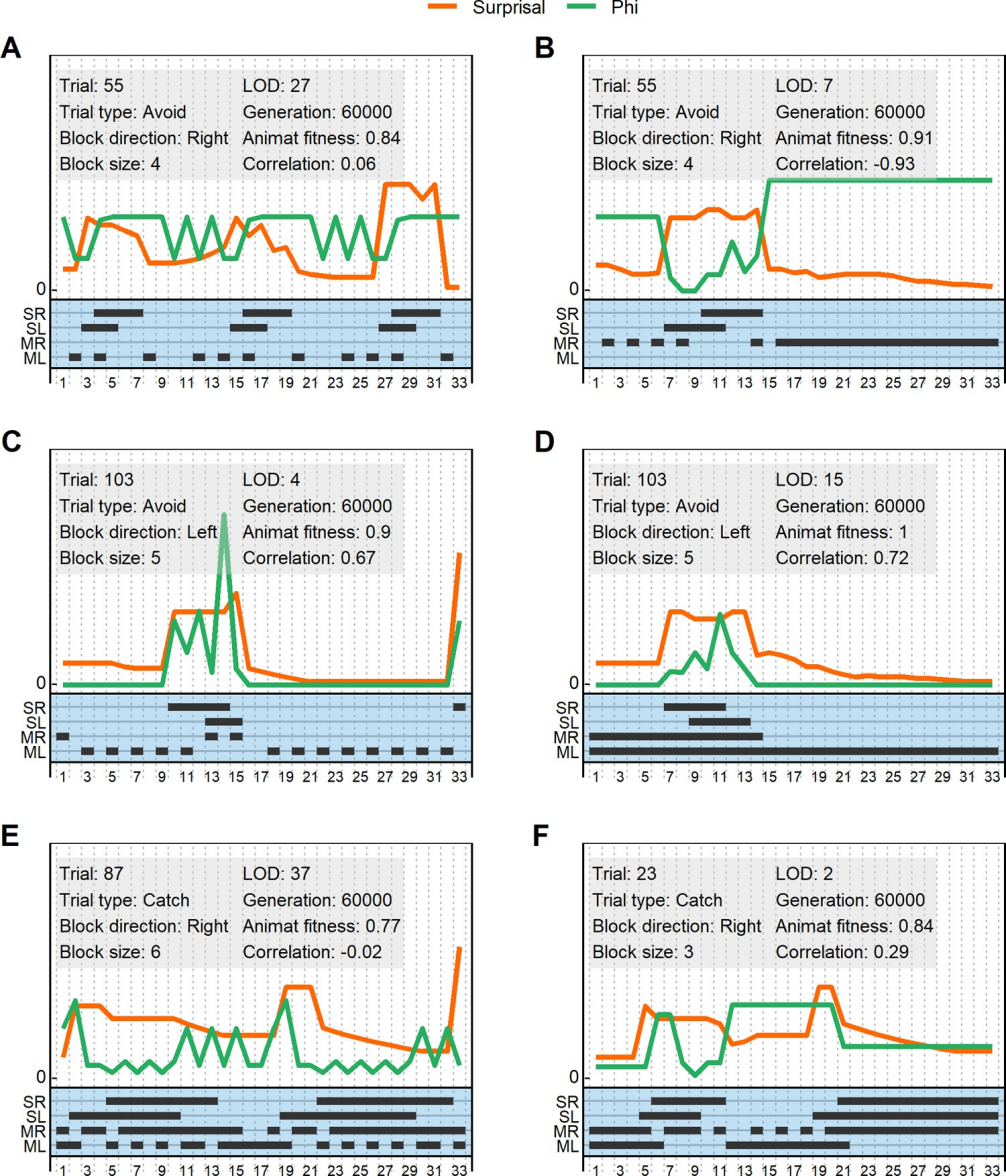
A-F) Within-trial representation of example animat behavior, Φ and surprisal. The gray box of each plot shows information about the trial and the animat, and the correlation between Φ and surprisal. The x-axis shows timesteps within the trial. The orange line denotes surprisal, and the green line denotes Φ. Each is plotted on an arbitrary y-axis for better comparison where only the zero point is indicated. The lower half of each plot shows the right and left sensory states (SR, SL) and motor states (MR, ML), with black lines indicating activation. Sensory states are active when the block is perceived. Motor states are activated by the animat, and makes it move in the given direction; if both motor states are active the animat stands still. Plots are chosen to show a variety of patterns of behavior, Φ and surprisal. All examples are taken from animats in the hard task.

Before presenting the correlation analysis, we show examples of per-timestep fluctuations between Φ and surprisal for a few individual trials (Fig 5). Here we see examples of different strategies for solving the task. The most common for adaptive animats is the “follow-strategy”, where animats follow either behind or underneath the block depending on whether they must catch or avoid it (Fig 5B and 5D and 5F). The second is the “pass-over-strategy”, where animats let the block pass over them, which can be equally adaptive in catch trials (Fig 5A and 5C and 5E; see Methods for visualizations of the behavior of perfect animats). These examples also exemplify the way that both Φ and surprisal often change at the times when the animat observes the block. Finally, we can see examples of positive correlations (Fig 5C and 5D and 5F), negative correlations (Fig 5B) and lack of correlations (Fig 5A and 5E) between Φ and surprisal for a particular single trial.

**Fig 6:**
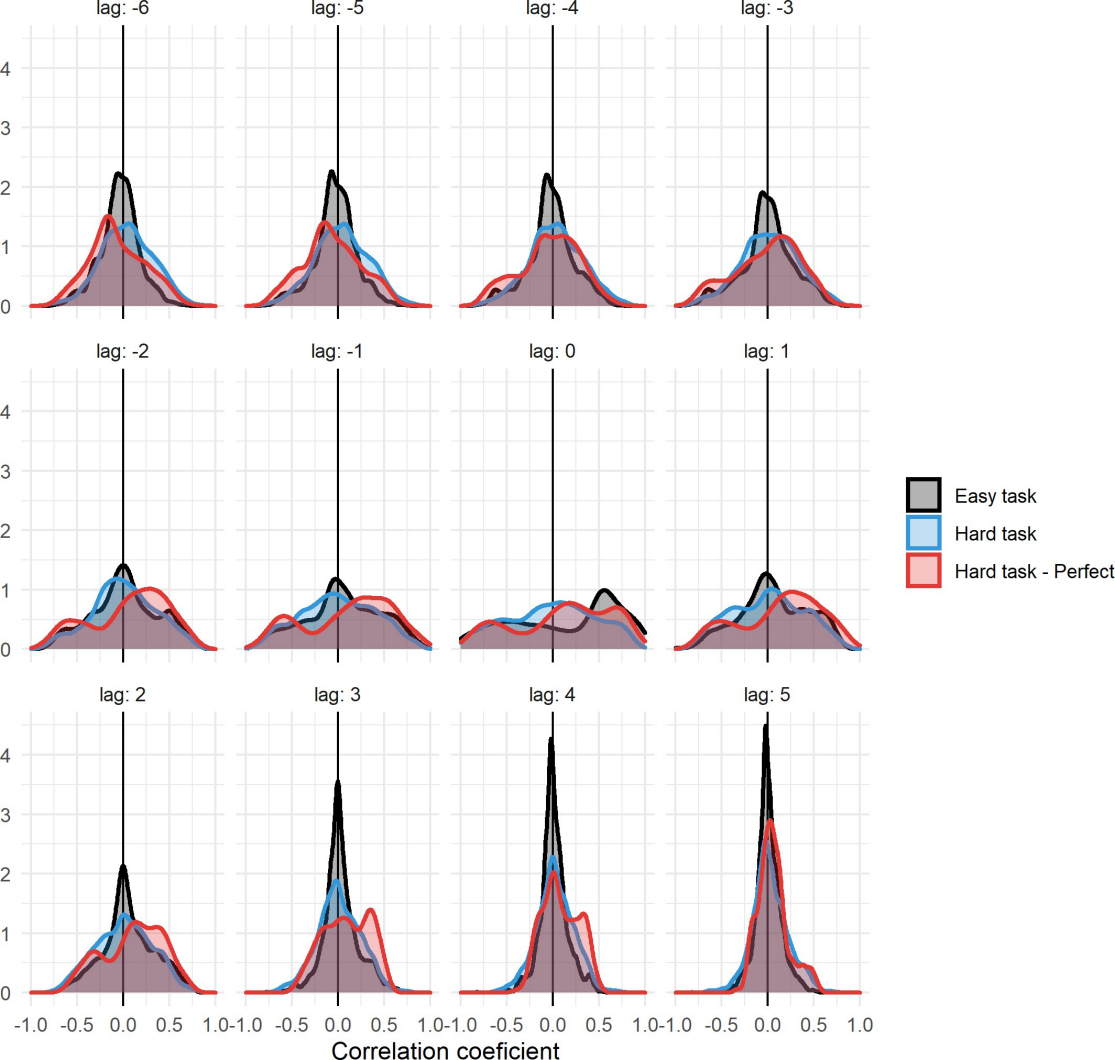
The distribution of correlation strengths between Φ and surprisal, across all trials on the last generation of all LODs. Shown for time lags around 0, denoted above each of the graphs. Correlations are shown for all animats in the easy task (black), for all animats in the hard task (blue) and for only perfect animats in the hard task (red). The lagging variable was Φ, meaning that for negative lags the correlations measure the relationship between Φ and future surprisal, and vice versa for positive lags. Note that trials where Φ = 0 throughout are excluded from this analysis.

Fig 6 shows the distribution of cross-correlations of Φ and surprisal across all trials of the final generation for all LOD’s. For time lags close to zero, there are many correlations that are far from 0 in both the positive and the negative direction, indicating that there is a relation between the two measures, but that it varies between trials and LODs. In general there is a positive skew for perfect animats in the hard task, and an even stronger positive skew for animats in the easy task. At larger time lags, correlation distributions are increasingly centered around 0, indicating that when Φ and surprisal correlate, they do so by fluctuating at the same times, as opposed to in a lagged manner. Additionally, a multilevel linear regression confirmed that there was a significant relation between the fluctuations (i.e., absolute change from last timestep) of Φ and surprisal at lag 0 (*β* = 0.123, SE = 0.013, p<0.001, random slope across animats (SD) = 0.018).

### Correlation profiles

In order to explore the mechanism behind the different directions of correlation (positive and negative) between Φ and surprisal, we here again present the results of the animats performing the hard task, but now split into three groups based on correlation profiles (negative, neutral and positive correlations between Φ and surprisal).

**Fig 7:**
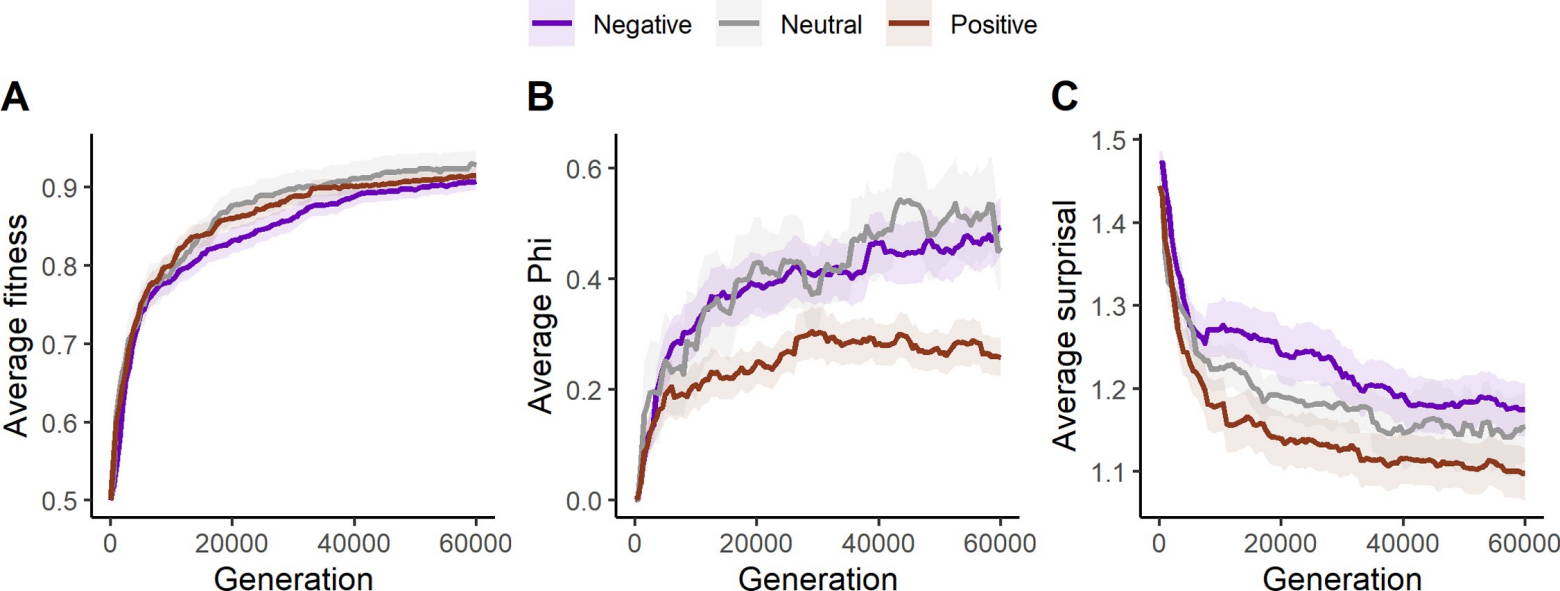
Change in fitness (A), Φ (B) and surprisal (C) over evolutionary time averaged across LODs sorted into groups depending on animat correlation profiles at last generation. The groups are: Negative (purple), neutral (grey) and positive (brown) correlations.

Fig 7 shows the results at evolutionary time. Here, the correlation profiles are based on the average correlation coefficients of animats at the end of evolution. The average fitness is similar in the three groups (Fig 7A), however, both Φ and surprisal are on average lower for animats with a positive profile (Fig 7B and 7C). We also see that animats with a negative profile seem to have slightly higher surprisal on average than those with a neutral profile, which again are slightly higher than the positive correlation profile animats.

**Fig 8:**
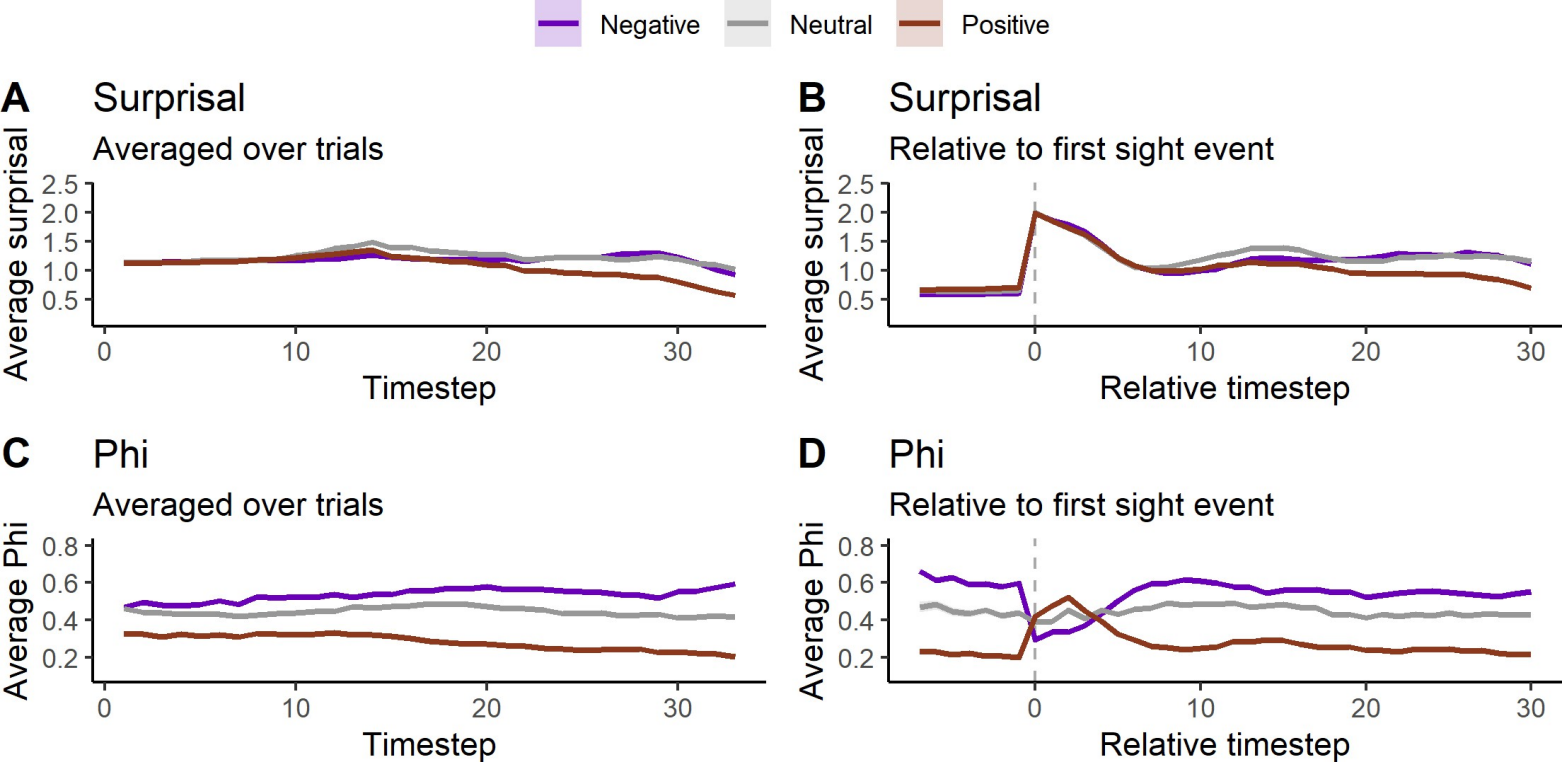
Average fluctuations in Φ and surprisal on trial time split into trials with negative (purple, coef. < -0.1, N = 4489), neutral (grey, coef. in (-0.1, 0.1), N = 1532) and positive (brown, coef. > 0.1, N = 5611) correlations between Φ and surprisal. A) average surprisal over trial time. B) average surprisal over trial time, centered around the first observation of the block. C) average Φ over trial time. D) average Φ over trial time, centered around the first observation of the block. Shading around lines is the standard error of the mean.

In Fig 8 we see the differences between correlation profiles at trial time. Here, the correlation profiles are based directly on the correlation coefficient at each trial separately. Since a given animat can have positive correlations on some trials, and negative on others, these plots are not comparable to the end point of evolution in Fig 7 before. Considering surprisal (Fig 8A and 8B), there is almost no difference between the groups, besides positive correlation trials having lower average surprisal towards the end. However, in Φ there is a much clearer difference between the groups (Fig 8C and 8D). The most striking difference is seen in Fig 8D where trial time is centered around the first observation of the block. We see that on trials with positive correlations, Φ is generally on a low baseline. Average Φ then rises when the block is observed, and falls again to a stable level. The pattern is the opposite on trials with negative correlations. Average Φ is the most similar between correlation profile groups in the period between the first block observation and the point at which average Φ returns to baseline level. This explains why we see no average fluctuation in Φ when including all animats in the hard task at the first block observation (Fig 4D), while still getting a broad distribution of correlations coefficients. The inverse patterns of positively and negatively correlated trials cancel each other out in the unified average. Additionally, the general difference of average Φ baseline levels between positive and negative correlation profiles explains the relatively big difference between these two groups in both evolutionary time (Fig 7B) and trial time (Fig 8C).

## Discussion

We evolved 50 animats in the easy task, out of which 34 reached perfect fitness, and 100 animats in the hard task, out of which only 8 reached perfect fitness (see Table 1). This shows that there is a substantial difference in difficulty between the tasks, which seems to affect Φ. Fewer animats showed no integration at the final generation in the hard task than in the easy task, and only 8.3% of all trials at the final generation had a mean Φ value of 0 in the hard task, whereas the easy task had a much higher percentage of such trials (22%). The maximum value of Φ found in the hard task (Φ = 4.11) was much higher than the maximum value found in the easy task (Φ = 2.49). There is a slight but clear difference in the distribution of Φ values at the final generation (Fig 2), seemingly driven by a difference in the number of zero-Φ states between the tasks, rather than a general level of higher Φ values. As such, it seems that the hard task puts more evolutionary pressure on the presence of integration rather than the level of integration, compared to the easy task.

On the evolutionary timescale we see similar results for Φ as in Albantakis et. al [47], although a considerably smaller difference between the tasks. Note that when using the median (see S1 and S2 and S3) the difference between tasks is much clearer, but there the perfect animats of the hard task are more similar to the rest of the group. The same is true for Φ-levels in trial time. There are both upper and lower bounds for Φ, so the deviations from low or high baselines upon observing the block might be due to a regression towards the mean, where animats with higher baselines on average decrease in Φ when its internal state changes, and vice versa. It is still interesting, and not a priori to be expected, however, that there is a baseline at all, and that animats seem to return to it after seeing the block.

In both evolutionary and trial time, the results regarding our surprisal measure were largely in line with expectations from the FEP. Average surprisal decreased as fitness increased over evolutionary time, which reflects the fact that average surprisal measures the difference from the empirically observed stably optimal behavior. Surprisal also increased at the time point when animats first observed the block; even perfect animats cannot control when this happens, which results in more variance and therefore more surprisal, and which also makes this the most informative time at a given trial, in a classic Shannon information sense. At the end of the trial, surprisal goes down on average, as animats become able to act in ways that make their sensorium more similar to that of adaptive agents, and less entropic as well. Surprisal is also lower in the easy task; that it is easier means that more animats successfully act in accordance with the empirical goal prior.

The results show a relation between Φ and surprisal. On the evolutionary timescale, this relation is likely due to the fact that both measures are related to fitness, so that as fitness increases, surprisal falls and Φ increases, resulting in a correlation between the ability to efficiently minimize surprise on one hand and on-average higher intrinsic causal integration on the other. On a within-trial timescale, we find that Φ and surprisal fluctuate at the same times, namely when the block is first observed by the animat. This provides some initial evidence for the claim that a change in (the surprisal of) sensory states for a system is related to a change in its intrinsic causal structure. Or, in other words and contingent upon both theories, that a change in sensation leads to a change in experience.

### Applying the Free Energy Principle

In order to relate our results to the Free Energy Principle, it is important to consider whether the animats fulfill the criteria for its application. The animats clearly and by construction possess stable Markov Blankets, composed of the sensory and motor nodes. However, they do not actively maintain their Markov Blankets through their own internal dynamics. Thus, it is harder to determine whether claiming they possess a non-equilibrium steady state is sensical and we will not try to do so formally here. However, empirically we observe that animat behavior converges towards stable patterns over evolutionary time, indicating a movement towards a NESS. Similarly, within a single trial, a successful animat also manages to enter a stable sensorimotor state of seeing or not seeing the block, depending on the task. Empirically, at least, then, animats seem to both possess a Markov Blanket and act in ways that make them enter and maintain an evolutionarily adaptive non-equilibrium steady state; it is indeed in these stable states where our surprisal measure is the lowest, further indicating that the measure is a good approximation of the generative-model based surprisal that is upper-bounded by variational free energy in the Free Energy Principle. This is useful, given that re-constructing the generative model implied by a given system’s actions, which is necessary for directly getting the variational free energy, is rarely easy.

Importantly, in a sense, the only probabilistic, continuous adaptation happening in our simulation happens on the evolutionary timescale, since animats are deterministic systems on the trial timescale. That raises the question of whether applying the Free Energy Principle, and the accompanying interpretation of the system as a probabilistic Bayesian belief updater, makes sense on the deterministic trial timescale. We will not make strong conclusions here, except to note that even though the animats are deterministic logic networks, they might still *look as if*, or be *well describable as if* they were doing probabilistic Bayesian inference. Indeed, it can be difficult not to interpret the animats’ block-identifying and -following behavior as goal-directed, planning based and inferential processes, despite knowing of their deterministic internal structure. In other words, the animats might behaviorally seem to be goal-directed Bayesian believers, even if they are just a simple network on the inside. This might seem like an unjustified anthropomorphization of a purely mechanical system, but should rather be read as an interpretation of the system’s functional relevance. Under the FEP, systems are not so much anthropomorphized as they are statisticomorphized, that is, deliberately interpreted as doing statistical inference. This might sometimes be a useful reframing of the mechanical system, partly because it can still be used when the entire internal structure of the system isn’t known, and partly because it lends a functional and goal-oriented interpretation to the behavior and structure of a network, which otherwise is just an arbitrary set of connected nodes to an outside observer. Here, the animats’ internal mechanistic structure is known in all detail. Moreover, multiple mechanistic implementations can lead to perfect fitness and similar behavior in the block catching task. Comparing a re-constructed generative model of individual animats to their actual mechanistic implementation may elucidate how generative models should be interpreted more generally.

### IIT and the FEP

One important question when relating IIT and the FEP is whether they are at all conceptually compatible. IIT is explicitly concerned with intrinsic physical-causal interactions (identifies conscious experience with an intrinsic physical-causal perspective). The conceptual stance when using the FEP is more varied: it is functionalist (occupied with abstract computational processes underwriting behavior) as opposed to physicalist; it is often instrumentalist (treats the computational constructs as a tool for describing behavior) with some advocating for a realist stance (where computations are considered real in their own right); and it is in important ways extrinsic (insofar as it is oriented towards representational relationships between internal states or beliefs and the external environment), although it at the same time stresses (more akin to an intrinsic account) that an agent’s beliefs and mind are technically only a function of the sensorimotor exchanges in the Markov Blanket that hides the real world from it. If one takes an instrumentalist view of the FEP, where the free energy minimization is thought of as a useful interpretation of a system’s behavior, the two approaches are clearly conceptually compatible. The theories might still be compatible under a realist interpretation, perhaps particularly if computational processes, which in IIT are normally not considered real, could be identified as causally integrated (and therefore real) mechanisms; however, some justifications for realist interpretations of the FEP often rest on extrinsic accounts of realism, as for example when based in representationalism [30] or contribution to objective goals [29]. Ontological commitments are in general stronger and more deliberate within IIT, which means that a conceptual compatibility between the two approaches might ultimately depend on which of the different possible ontological stances compatible with the FEP one chooses to commit to.

An obvious, and important, empirical question which becomes apparent when relating the two frameworks is then: is the intrinsic experience of a system homomorphic to the statistical beliefs implicit in its behavior? This, or even a complete identity between the two, is often casually assumed by functionalist theories of consciousness. However this, at least in theory and perhaps also in practice, does not always need to be the case. According to IIT, systems with different internal causal structures, and therefore different intrinsic experiences, can be functionally identical [2], and therefore seem to hold the same Bayesian beliefs. However, there might be good reason to believe that, at least most of the time, intrinsic experience and functionally implicit statistical beliefs are closely related. Systems that seem functionally identical in a given context can have widely different circumstantial constraints, such as energy cost, because of different internal structures, meaning that they would not be equally able to keep existing in a given environment. Apart from making such systems functionally heterogeneous on this longer timescale, this suggests that the more efficient solution (e.g. lowest energy consumption) will probably be the most likely, reducing the amount of functionally identical but internally different systems that in practice exists in nature. FEP’s findings that internal structures of systems come to resemble the environment [20,51] also indicate that systems in shared environments might come to have similar intrinsic experiences. However, that the animats in our study show different baseline levels of Φ, despite evolving in identical environments and with identical constraints, suggests that the intrinsic causal structures of such integrated systems can vary in ways potentially substantial for the quality of experience (although it is hard to assess the magnitude of differences in Φ values).

IIT does not inherently claim that intrinsic experience will necessarily depend directly on sensory inputs. However, sensory inputs contribute to determining the internal state of the system, which in turn determines the system’s state-dependent causal structure and Φ value. Under the FEP, more specifically, sensory observations should affect the configuration of internal states in ways that reflect the most probable cause of the observation, which in turn might facilitate a stronger relation between contextualized sensory inputs and intrinsic experiences. In our simulation, we see initial evidence for this: Φ indeed fluctuates when the observation of the block affects the internal states (which should also be the time when there is high extrinsic information content of the animat’s sensory states).

### Venues for further work

The relationship between intrinsic experience and adaptive behavior guided by implicit statistical beliefs is difficult and important, but is often casually assumed or ignored by modern cognitive science and neuroscience. Working with both the FEP and IIT might help elucidate this relation. One obvious direction is to get a generative model for an active inference agent where Φ can also be calculated. This would first require constructing an active inference model which can reproduce the behavior of the animats on the block catching task. Given that the environment, action space and time are all discrete, this can be done as a Markov Decision Process (MDP) model. Subjecting such an optimal active inference agent to the exact same sensorimotor exchanges as a given animat would then provide the optimal Bayesian beliefs, precisions and variational free energy of an active inference agent in that situation, which can then be compared to Φ and the conceptual structure. It is also an option to fit such MDP-based active inference models to the behavior of the animats, as one might to a human participant (as in [31]). Comparing different models like this would allow for inferring which animats seem to have which model structures, and for example correlate Φ with having a conceptual structure with a longer temporal and counterfactual depth (as is suggested by Karl Friston [36]), which seems compatible with the fact that the more advanced animat behaviors indeed display an ability to distinguish between more contexts, and to plan further ahead in time.

In general, the work to relate the FEP and IIT, and probably much of consciousness science and neuroscience in general, would benefit greatly from some thorough conceptual work clarifying the relations between the terms and concepts in the different theories. Words such as complexity, intrinsicality, and ‘thing’ are used with potentially different meanings, or at least different operationalizations, in the two theories. There is much to do in terms of investigating the conceptual and formal relations between the constructs in IIT and FEP, but this comes with potential for great gains. As an example, there might be in practice a relationship between a stable Markov Blanket and the borders of a system’s main complex—perhaps maintaining a causal border like the Markov Blanket also often results in complexes not stretching beyond that border. Perhaps not. The relation between extrinsic and intrinsic causal borders, that is, what it means to be a thing to the outside observer, and what it means to be a thing to oneself, is so far not clear. In general there is much work to be done in order to bridge the two frameworks, not to mention the colossal work being done within each framework to further develop and improve them separately.

## Methods

### Simulation details

The simulation was done using the simulation framework MABE [52]. This framework, and the particular settings and environment implemented, have been used to study IIT in an evolutionary context before [53]. We base our study on the code used in [47]; all code and simulated data can be found on a time-stamped repository at osf.io/uzpca.

The task environment was a two-dimensional space that had a width of 16 square units and a height of 36 square units. The left and right side of the environment were connected, so if something moved across the edge of the environment it would appear on the opposite side. On each trial, a horizontal block of varying length would move across the task environment in a series of timesteps. On each timestep the block would move one unit down and one unit to the side until it reached the bottom of the task environment. Blocks would move consistently and unidirectionally during each trial reaching the bottom in 35 timesteps. The first timestep, where the animats are initialized in the same all-off state, and the last timestep, where animats are no longer performing the task but instead enters a win-or-lose state, were not used, meaning that the analyzed trials consisted of 33 timesteps.

Within the environment, an animat is represented as a three unit block at the bottom of the task environment. It constituted a small Markov brain [49] consisting of two sensory nodes, two motor nodes and four hidden nodes, all with two possible states (on and off). The animats’ sensors were positioned on the outermost units of the animat block. They would activate the corresponding sensory nodes if a block was in a direct line above it. The animat would then move left or right when the corresponding motor node was activated. If both motor nodes were in the same state, either on or off, the animat would not move. Within the network of the animat’s Markov brain it was possible to form connections between all nodes, except that sensory nodes could not have inputs and motor nodes could not have outputs. The hidden nodes and the motor nodes would activate based on inputs according to a specific logic, which is also adapted during evolution.

When the falling block reached the bottom of the task environment, it would be considered a catch if the animat was overlapping with the block, otherwise the animat would have avoided the block. The task was for the animat to catch blocks of a certain length and avoid blocks of other lengths. In [47] there were 4 different task conditions, but here we only focus on task 1 (block lengths 1: catch and 3: avoid) and task 4 (block lengths 3, 6: catch, and 4, 5: avoid), which in our paper is called the “easy task” and the “hard task” for simplicity.

150 evolutionary runs were simulated. Each run consisted of 60,000 generations and each generation of 100 animats. In the first generation, the animats had no connections between their system nodes. After each generation, the animats of the next generation were sampled from the animats of the current generation with replacement. The better an animat performed, the more it was sampled compared to other animats. After the animats of the next generation had been sampled they would mutate according to a genetic algorithm, resulting in changes in the nodes’ internal logic and their connections. For each run, data was recorded on the best performing animat’s line of descent (LOD) every 500 generations. Thus, 121 animats were recorded for each of the 150 LODs. Each animat performed 128 trials, each of 33 timesteps (excluding the first and the last timestep). Depending on the task difficulty, the animat would encounter two or four blocks of different lengths. Out of the 150 simulation runs, there were 50 runs of the easy task, which is the same number of simulations as in [47]. However, we simulated 100 runs of the hard task, in order to both get a better sample size when grouping the data and get a larger number of perfect animats. For more details on the simulation environment and the evolutionary algorithm, see the methods section of [47].

### Calculating Φ

**Fig 9:**
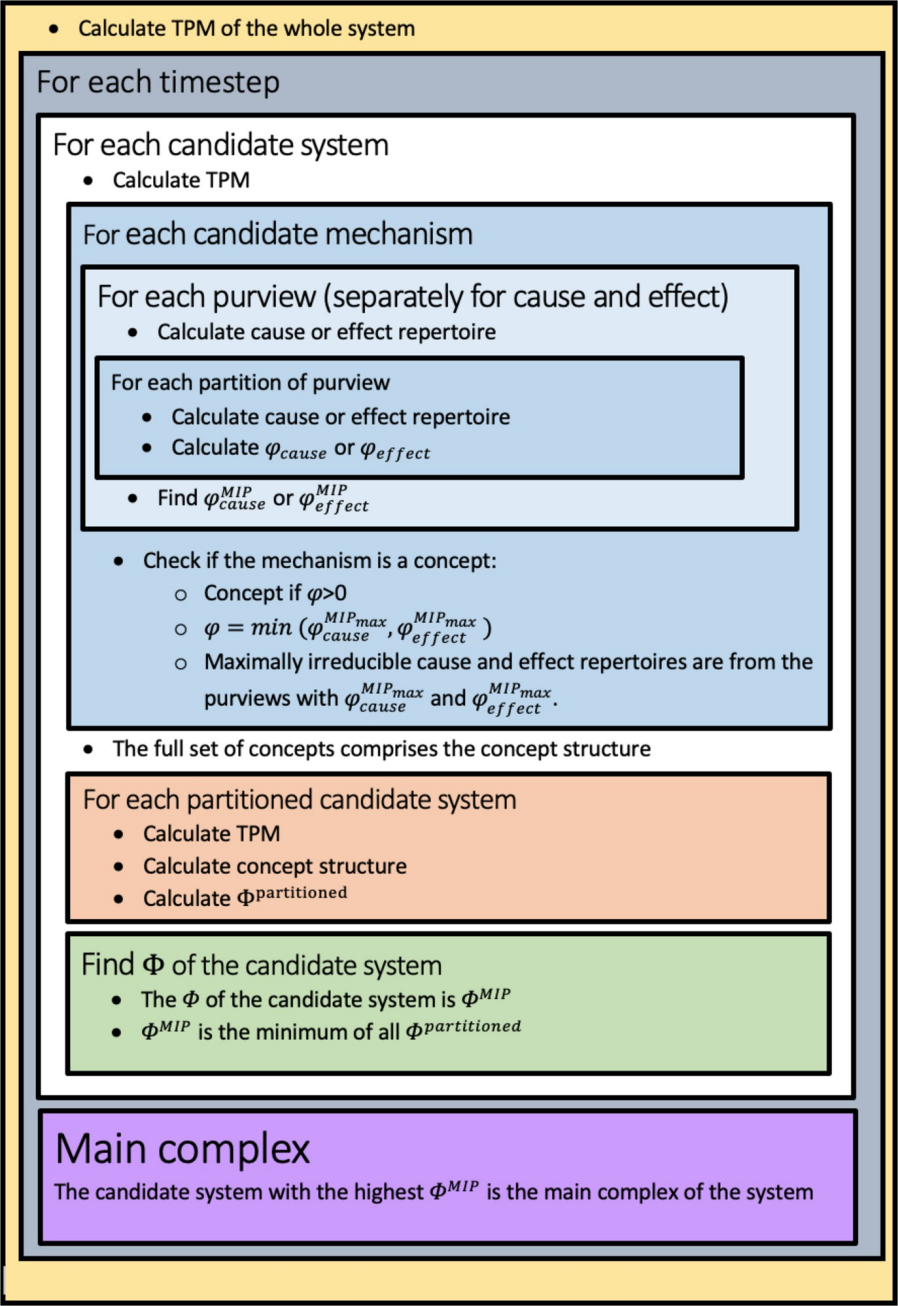
A summary of the procedure for calculating Φ.

The IIT analysis was carried out in python using the PyPhi package [54]. Here we use the third iteration of the IIT formalism (“IIT 3.0”) as described in [2]. The following section will describe in short the procedure for calculating Φ, summarized in Fig 9. At the moment, calculating Φ is only possible for discrete systems in discrete time [55]. Φ must be calculated for each timestep of interest, and thus, is state dependent. First a Transition Probability Matrix (TPM) is made, representing the internal logic of the system’s elements. Every set of elements within the larger system are then evaluated as candidate systems, including the whole system. Elements outside each candidate system are called background conditions and are fixed in their current state during the analysis of each specific candidate system. Integrated conceptual information Φ is then calculated for each candidate system, where the candidate system with the highest Φ is the system’s main complex. In principle, non-overlapping sets of elements may form additional complexes that would also be considered conscious.

When analyzing a candidate system, one first derives its TPM from the full system’s TPM. From the candidate system’s TPM one can calculate its unconstrained cause and effect repertoires. A cause repertoire is a (product) probability distribution across system states at the previous timestep, the probability of each state being the probability that it preceded the current state (for details see [2]). An effect repertoire is a (product) probability distribution of how likely system states at the following timepoint are to occur given the current state. The unconstrained cause and effect repertoire are calculated given no information about the current state.

Next, the powerset of candidate mechanisms are found, i.e. all possible subsets of the candidate system including the whole system. Then for each candidate mechanism all possible purviews of the mechanism are evaluated. A purview consists of a mechanism at the current timestep and the same or a different set of elements at the previous timestep (cause purview) or the next timestep (effect purview). The purviews are evaluated in order to find the purviews over which a maximum of integrated information φ is specified for the cause and effect separately (φ_cause_ and φ_effect_). φ is calculated as the earth mover’s distance between the cause or effect repertoire of the purview (the probability distribution given the purview) and the partitioned cause or effect purview under the minimum information partition (with the minimum distance to the repertoire of the non-partitioned purview). The minimum information partition is found by iterating over all possible partitions and calculating this distance. The sets of elements pertaining to the cause and effect purviews that specify a maximum of φ are the mechanism’s core cause or core effect, respectively. The overall φ value of the mechanism itself is the minimum of φ_cause_ and φ_effect_. In the case that this is a non-zero value, the mechanism and its cause-effect repertoire constitute a concept.

When all concepts of the candidate system are found, these concepts constitute a conceptual structure. To calculate integrated conceptual information Φ, the candidate system is partitioned in all possible ways. The partitioning is now unidirectional, so that either the input or the output of an element can be cut, but not both. For each of the partitioned systems, the conceptual structure is derived. Φ is then calculated as the distance between the conceptual structure of the non-partitioned system and the conceptual structure of the system under the minimum information partition, using an extended version of the earth mover’s distance. Again, the minimum information partition here is the way to partitioning the system, such that there is a minimum distance. Note that both here and at the level of mechanisms, using the minimum information partition quantifies how much the mechanism or the system cannot be reduced to its parts and thus how much it is integrated to be “something” above and beyond its parts.

### Approximating surprisal

In order to calculate surprisal, we first construct *empirical goal priors*, an empirical approximation of the expected sensory states an adaptive animat would have. Based on this probability distribution, we calculate the surprisal at each timestep in each trial for each animat.

Specifically, the empirical goal prior is the probability distribution over sensory states observed in perfect animats at the end of evolution (with four possible states being the four constellations of either active or inactive sensory nodes). The distribution is created by counting how often each sensory state occurs across trials on the final generation for all animats with perfect fitness. Separate probability distributions are created for each perfect animat, for each combination of task type (catch or avoid) and block movement (left or right), and for each timestep during the trial. We make separate probability distributions for each perfect animat because there are multiple optimal strategies, so that averaging across them would result in probability distributions that do not appear for any one animat. Similarly, we create different probability distributions for different task types and block directions because these contextual conditions result in different kinds of sensory states being related to adaptive behavior. Finally, we make different probability distributions for each timestep because there are qualitatively different patterns of sensory states in the beginning of a trial, compared to the end of a trial, where adaptive animats are able to converge on a pattern of sensory states. We do not distinguish between different block starting positions, nor between different block lengths within the same trial type, as these do not affect the kind of observations that are related to adaptive behavior.

**Fig 10:**
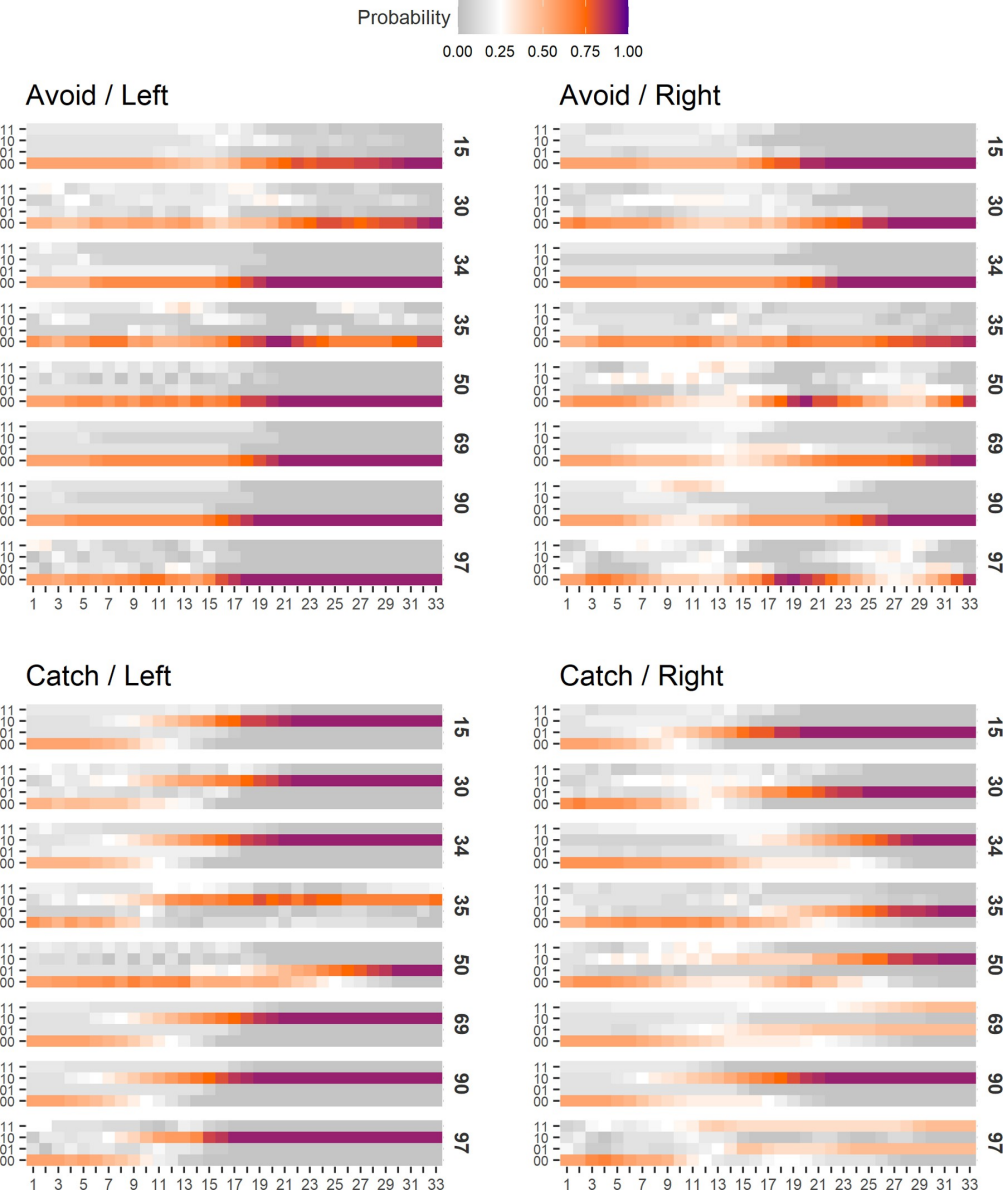
Empirical goal priors (EGPs) of the LODs in the hard task, which reached perfect fitness. For each perfect animat, 4 EGPs were constructed based on distinguishing first, catch and avoid trials, but also, left and right trials. The top row of blocks represent the avoid EGPs and the bottom row the catch EGPs. Those based on left trials are on the left and those based on the right trials are on the right. Each block consists of 8 heat maps, one for each of the LODs. The heat maps have 33 columns, one for each timestep (minus the first and last), and 4 rows, one for each possible sensory state. The sensory states are denoted by two binary digits, the first representing the left sensor and the second the right sensor (0 = off, 1 = on). Each cell in the heatmap represents the probability of the given sensory state at the given timestep. The color coding is constructed such that white represents a 0.25 probability. As the probability decreases below 0.25 colors become increasingly gray, and as the probability increases above 0.25 colors become increasingly orange. Probabilities with a value of 1 have a dark purple color to indicate states of certainty.

Surprisal is then calculated as the negative log probability of a given sensory state occurring given the timestep, task type and block direction:

ℑ(o)=−ln(P(o))

with ℑ being the surprisal, *o* the observations and *P(o)* the empirically observed probability over sensory states, given a timestep, a task type and a block direction. Surprisal is calculated separately for each of the probability distributions belonging to each of the perfect animats. The distribution which results in the lowest average surprisal for one animat at one generation across trials is then used as its surprisal score at each timestep, ensuring that surprisal is calculated relative to the perfect strategy which is the closest to the strategy employed by that animat. Fig 10 shows depictions of these goal priors, relative to which the empirical surprise is calculated. Here we see sensory state probability distributions for each of the perfect animats, in each of the four types of trials, over trial time. We see how goal priors become much less entropic as trials end, because animats are better able to act in ways that ensure their sensorium remains in a state that is associated with adaptive behavior. We also clearly see the different strategies employed by the animats: most animats use the following strategy, where they either see or do not see the block (depending on task type) for the entirety of the trial after inferring its size; or they use a pass-over strategy where they see the block twice. Technically, the goal prior is meant to be an evolutionarily selected expectation for receiving sensory inputs corresponding to self-maintaining and self-replicating behavior. In this example, that would be an expectation for succeeding on trials, from which expectations for catching or following blocks would follow, in turn giving rise to expectations of specific sensory-motor patterns. Our empirical goal priors, however, are defined directly for sensory states, and are not technically expectations on what is relevant for survival (success on tasks). Nevertheless, they implicitly are expectations for trial success, given that they are expectations for behavior observed in the agents which perform the task perfectly.

These empirical goal priors are used to calculate an approximation of the surprisal that would be associated with an animat encountering a given set of sensory states, if it were to be performing active inference under a generative model of its environment. Such a model is simply a probabilistic specification of the relations between environment states, sensory states and animat action states: *P*(*o*, *s*, *u*, *θ*). Here, *o* are the animat’s observations (the sensory states), *s* are the states of the environment (block length, block direction, block position, own position etc.), *u* are the active states of the animat (moving left or right or standing still), and *θ* are the probabilistic relations between each of these, the parameters of the generative model. Note that *s* and *u* would be inferred by an animat at within-trial time, while the parameters *θ* would be learnt over evolutionary time. Note also that the generative model would not necessarily correspond exactly to the generative process in the environment, since models in addition to becoming more accurate also become simpler with free energy minimization, abstracting away irrelevant parts of the environment.

The marginalization of *P*(*o*, *s*, *u*, *θ*) required to produce *P*(*o*) is in most cases computationally intractable. This is the reason that a variational free energy upper bound is used instead, forming the heart of active inference under the free energy principle. We here circumvent this step by approximating *P*(*o*) empirically. This allows us to investigate the animats’ surprisal without reconstructing their generative models *P*(*o*, *s*, *u*, *θ*), but also distances us somewhat from the Free Energy Principle in two ways. Firstly, the approximation is not necessarily perfect. The actual generative models that best account for the animats’ behavior might vary, either in their goal priors, so that some animats might expect to fail the task, or in how they expect states of the environment to interact, so that the animat might have bad predictions about the consequences of its actions. The former case would of course be removed by evolution, and the latter case is exactly what distinguishes adaptive from non-adaptive animats, for those animats with ill-specified generative models will often have incorrect expectations, leading to higher surprisal despite their attempts at minimizing it. The second way in which we depart from the Free Energy Principle is that the variational free energy is an upper bound on surprisal, where the difference between variational free energy and surprisal is the divergence from the animat’s current model of the world, relative to the Bayes-optimal model given the sensorium. The difference between surprisal and the variational free energy is also not captured by our approximation; this is not necessarily a problem, however, since the goal of free energy minimization in the FEP is after all the implicit minimization of surprisal.

It is also worth noting that there exist other formal frameworks for describing and producing task-optimal behavior, and that some of these may also be relatable to our surprisal measure. Perhaps most relevant is KL-control, a control-theoretic approach where an agent chooses actions that minimize the divergence between a target and an expected distribution of (sensed or inferred) states [56]. Active inference generalizes KL-control by additionally minimizing the ambiguity of sensory observations [25], but since there is no ambiguity in the current task, behavior based in KL-control and in active inference is likely to be very similar. This should mean that our surprisal measure is likely to also reflect aspects of KL-control like the expected control cost. This construct, however, differs from our surprisal measure in it not being linked to making sensory observations, but rather being a component of action planning and selection. More importantly, while active inference is grounded in the FEP, and is used as a theoretical framework for understanding self-organization and life generally, control theoretical approaches more commonly are used in machine learning and engineering contexts. Our interest here is to investigate the relationship between IIT as a theory of consciousness and the FEP as a cognitively interpretable account of adaptive behavior grounded in self-organization (as opposed to an approach for machine learning that may or may not be useful). Our focus is thus on surprisal in its capacity as a proxy measure reflecting the FEP, and not on how it might relate to control-theoretical approaches like KL-control.

### Computational analysis of the relation between Φ and surprisal

The computational analyses beyond calculating Φ and surprisal, as well as the production of all plots, was coded in the programming language R [57]. All analysis scripts can be found at the project’s OSF page: osf.io/uzpca.

The cross-correlation analysis was carried out using the ccf function of the tsibble package [58]. Cross-correlations with lags ranging between -16 and 16 were calculated for each trial of all last generation animats (see S4 for distributions across all lags). Since the correlation coefficient can’t be correlated when one of the variables is always 0, all trials with a constant Φ value of 0 were excluded (24.8% of trials in the easy task and 8.9% of trials in the hard task). The lagging variable was Φ, meaning that for negative lags the correlations measure the relationship between Φ and future surprisal, and vice versa for positive lags. Correlation profiles were derived from the 0 lag correlation coefficients. Neutral profiles had correlation coefficients between -0.1 and 0.1, negative profiles had coefficients below that range and positive profiles above it. For analysis in trial time each trial was assigned a correlation profile independent of what LOD or animat it belonged to. For analysis on evolutionary time each LOD was assigned a profile based on the average correlation coefficient across all trials of the final generation animat.

We performed a multilevel linear regression to test for a correlation at the last generation between fluctuations (i.e., absolute change from last timestep) in Φ and surprisal at lag 0, with random intercept for animat *a* and trial *τ*, as well as a random slope for animat *a*. Here, the first timestep of each trial was excluded as a change from last timestep cannot be calculated at the beginning of the trial.


$$\left|\Delta \Phi |\;=\;\beta_{0a,\tau }\;+\;\beta_{1a}\cdot |\Delta ℑ\right|$$

For the analysis in trial time, only trials belonging to animats on the final generation were used. For the analysis in evolutionary time, all Φ and surprisal values were first averaged across all timesteps for each animat to create an evolutionary time series for each LOD. These were smoothed by averaging each generation with the previous 5 generations. This was done in order to resemble the methods in [47]. Then all LODs were averaged across each generation to create the average evolutionary run. The whole process was done separately for each group. Although the animats of the hard task ending with perfect fitness were a group on their own throughout the analyses, they were also included in the general group of hard task animats.

## Supporting information

S1 Fig Median version of Fig 3. Versions of core figures in the text using median instead of mean values.
(PDF)

S2 Fig Median version of Fig 4. Versions of core figures in the text using median instead of mean values.
(PDF)

S3 Fig Median version of Fig 8. Versions of core figures in the text using median instead of mean values.
(PDF)

S4 Fig Correlation plot with all lags. Cross-correlation plot between surprisal and Φ, with all lag sizes.
(PDF)

S5 Fig Trial time fluctuations split separately for LOD’s. Trial-time fluctuations shown with each line of descent displayed separately.
(PDF)

S6 Fig Average relative Phi for each animat. Trial-time fluctuations shown with each line of descent displayed separately.
(PDF)

S7 Fig Average relative surprisal for each animat. Trial-time fluctuations shown with each line of descent displayed separately.
(PDF)
