# Supplementary figures and images for: Phi fluctuates with surprisal: An empirical pre-study for the synthesis of the free energy principle and integrated information theory
Source: PLoS Comput Biol. 2023 Oct 20;19(10):e1011346. doi: 10.1371/journal.pcbi.1011346 (PMC10619809; doi:10.1371/journal.pcbi.1011346)

Easy task    Hard task    Hard task - Perfect

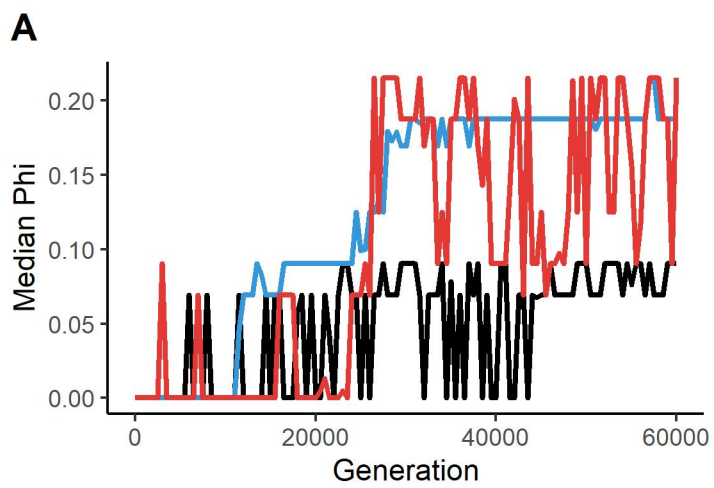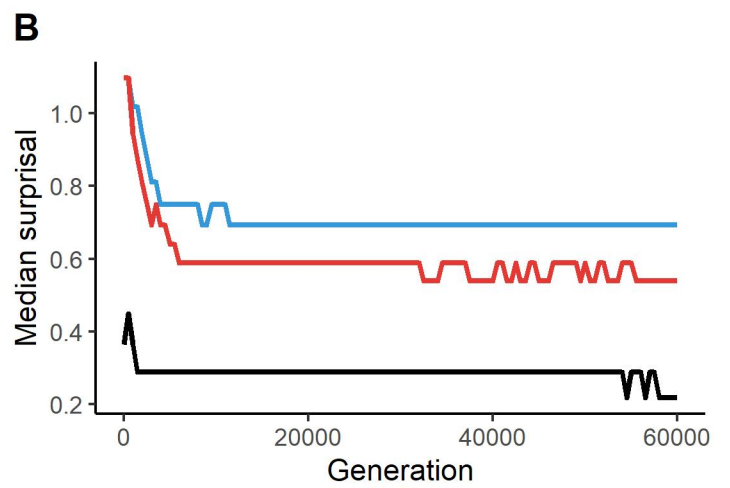

Supplement: S1 Fig — Versions of core figures in the text using median instead of mean values. (PDF) [file pcbi.1011346.s001.pdf]

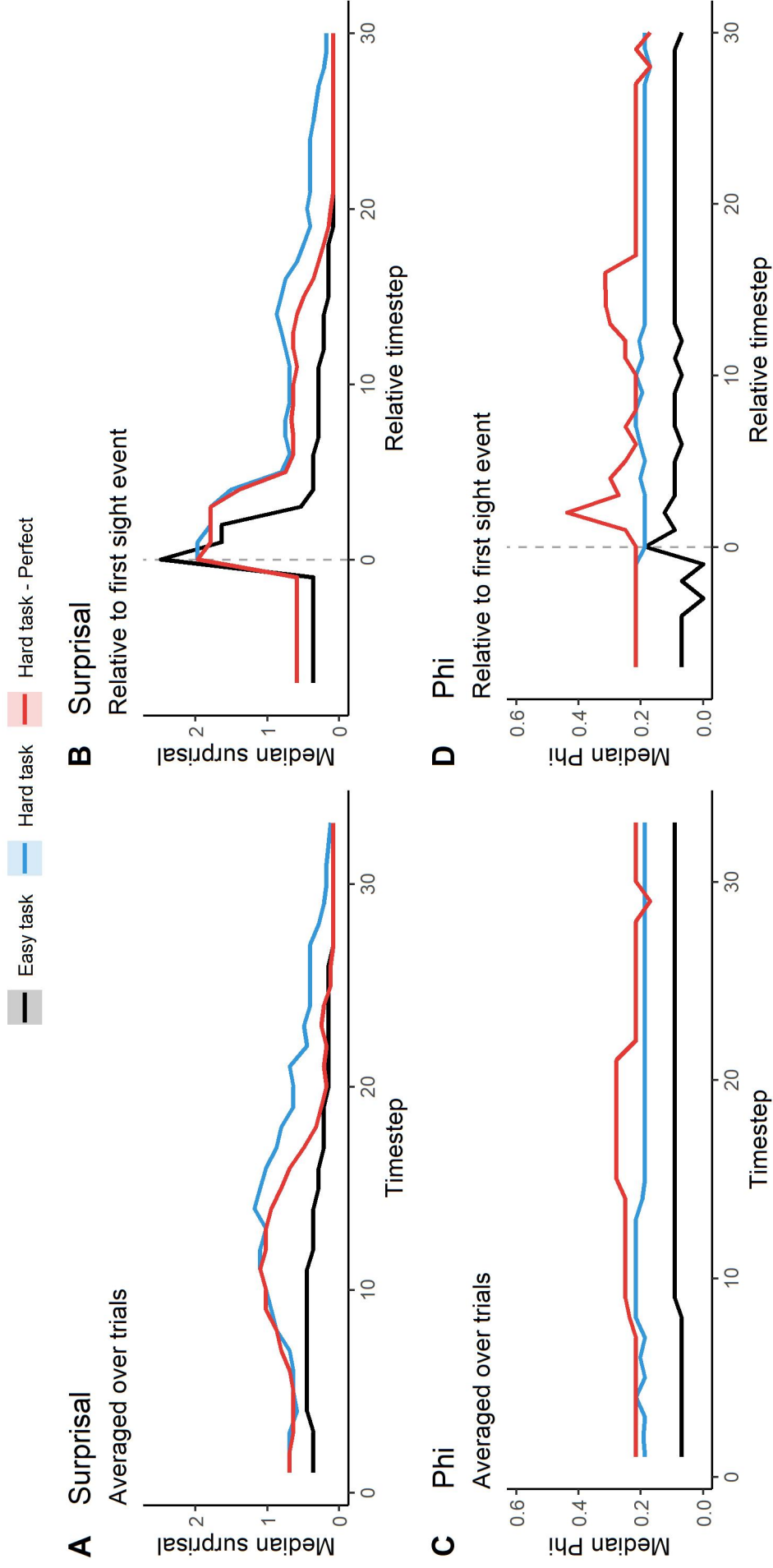

Supplement: S2 Fig — Versions of core figures in the text using median instead of mean values. (PDF) [file pcbi.1011346.s002.pdf]

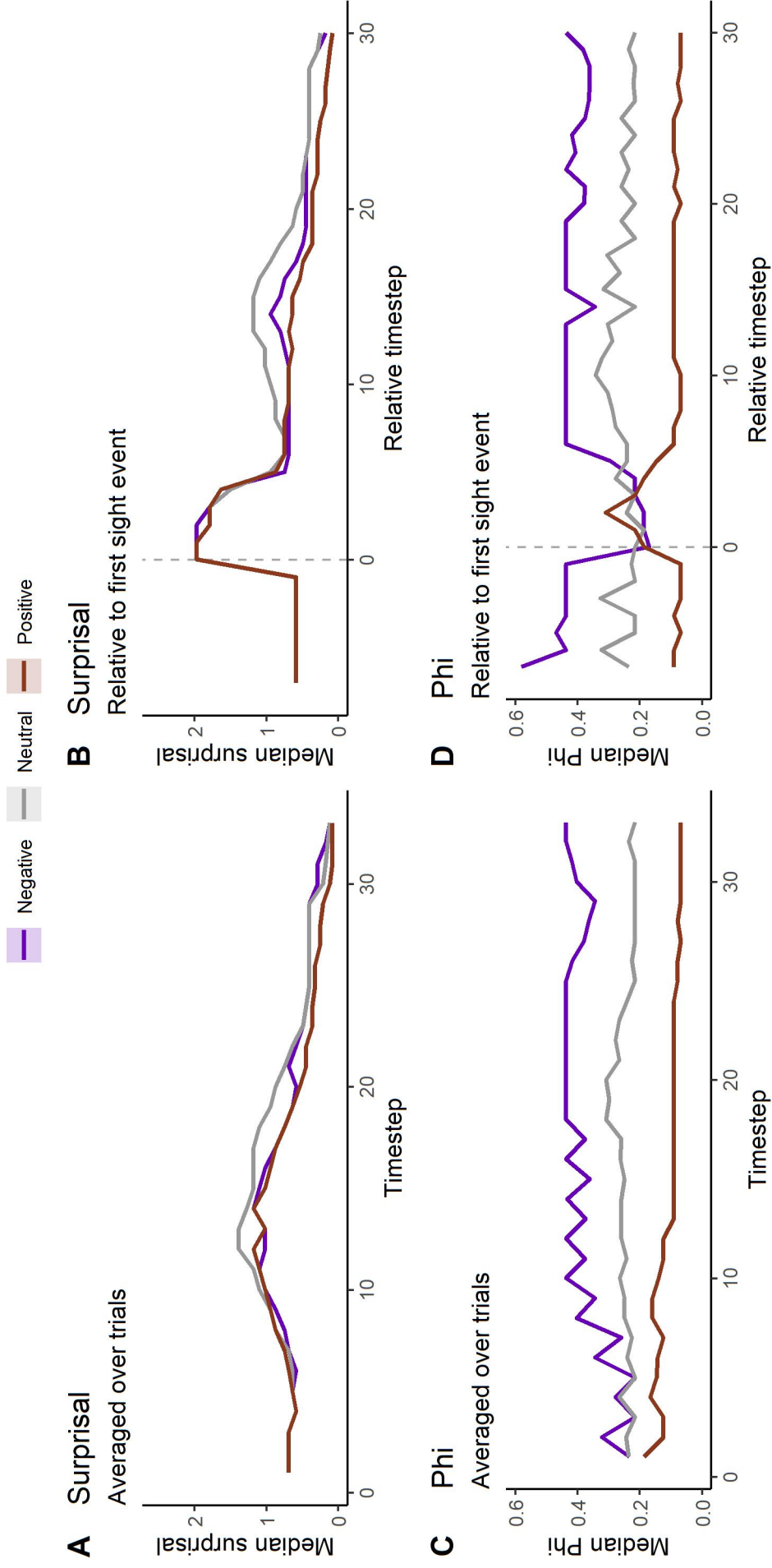

Supplement: S3 Fig — Versions of core figures in the text using median instead of mean values. (PDF) [file pcbi.1011346.s003.pdf]

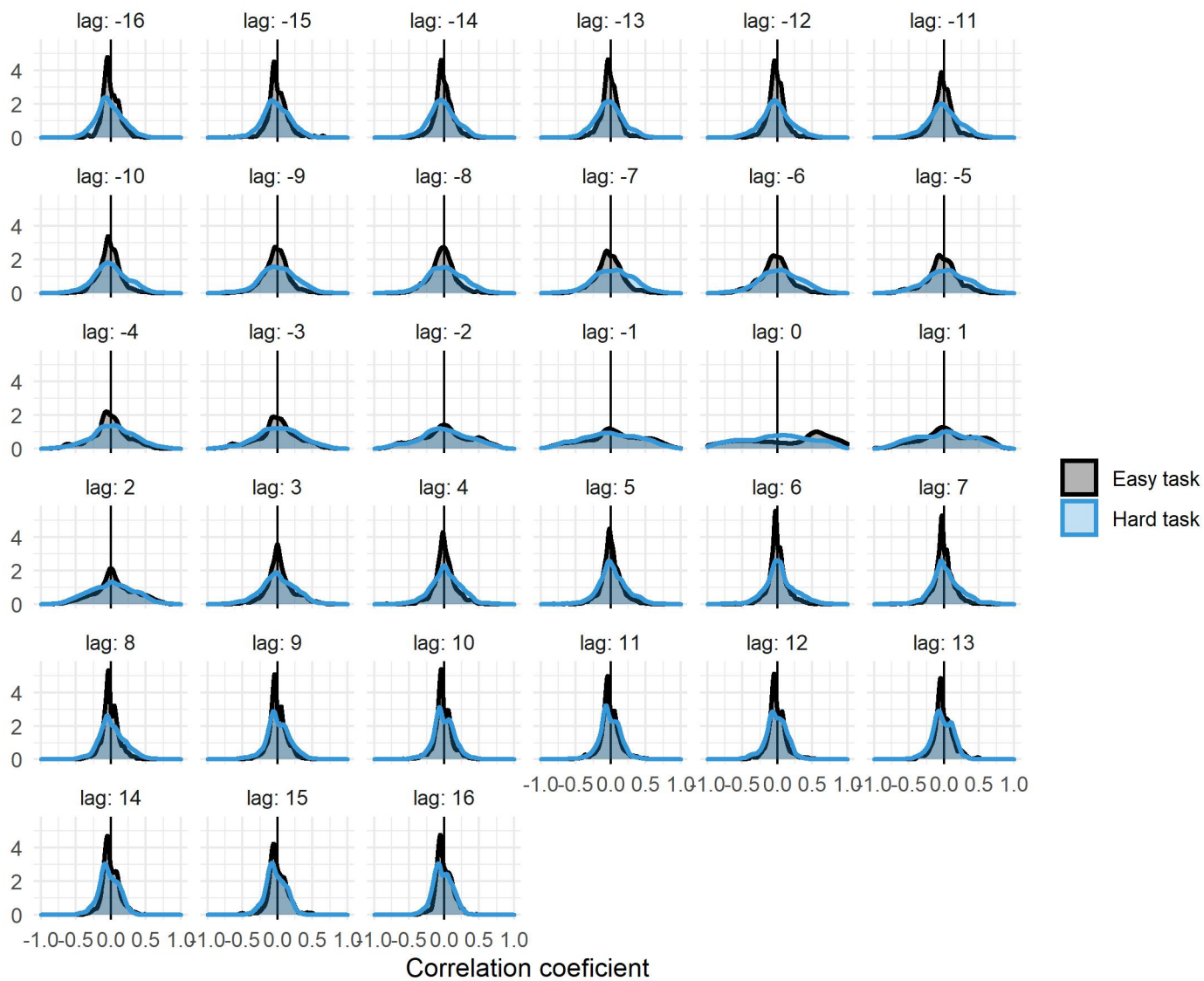

Supplement: S4 Fig — Cross-correlation plot between surprisal and Φ, with all lag sizes. (PDF) [file pcbi.1011346.s004.pdf]

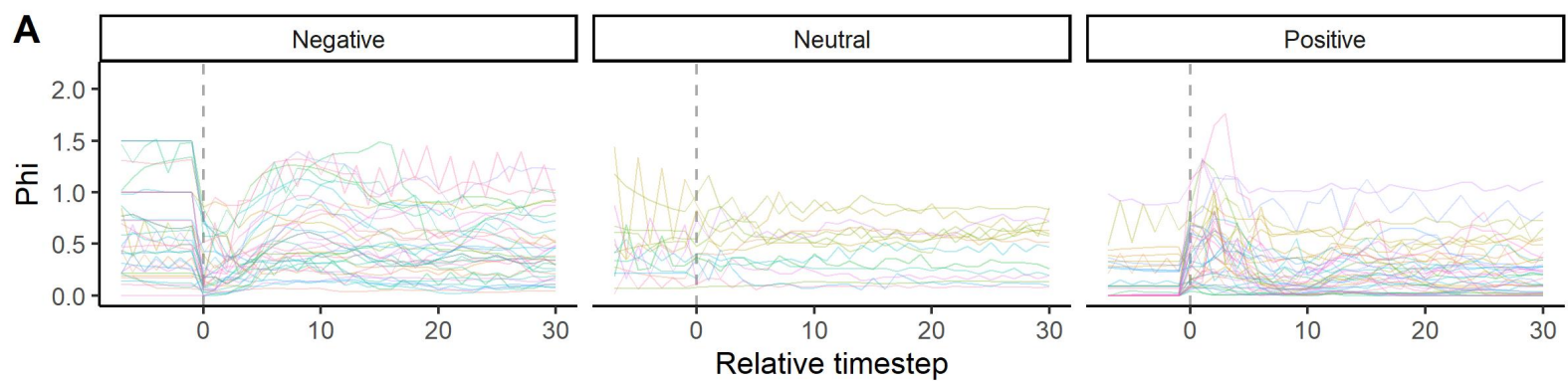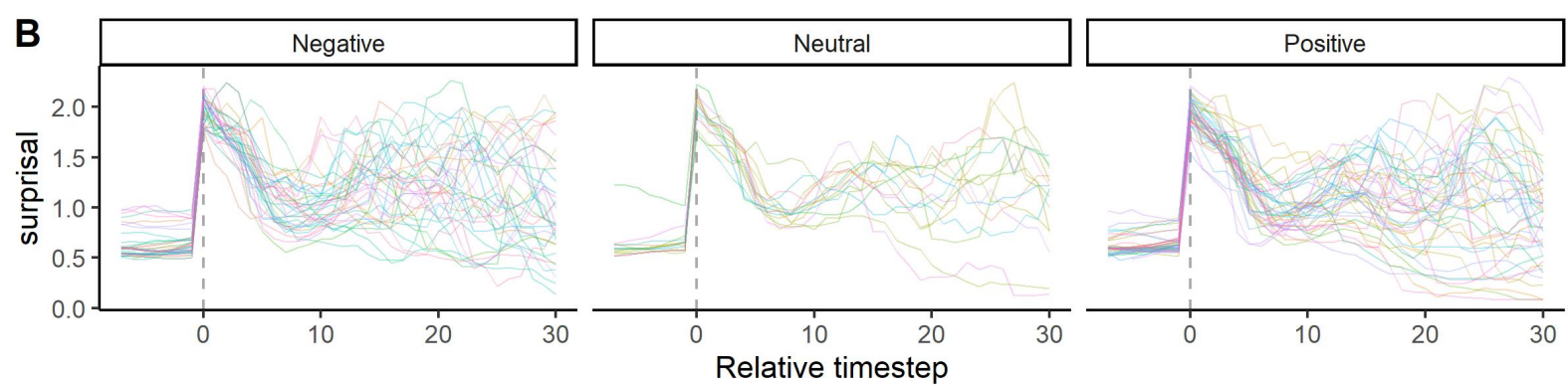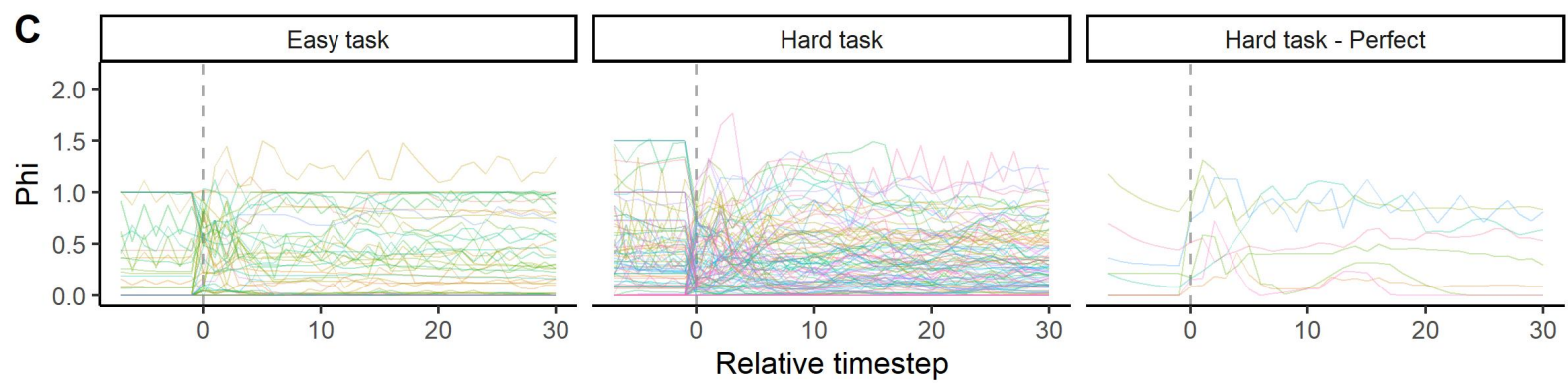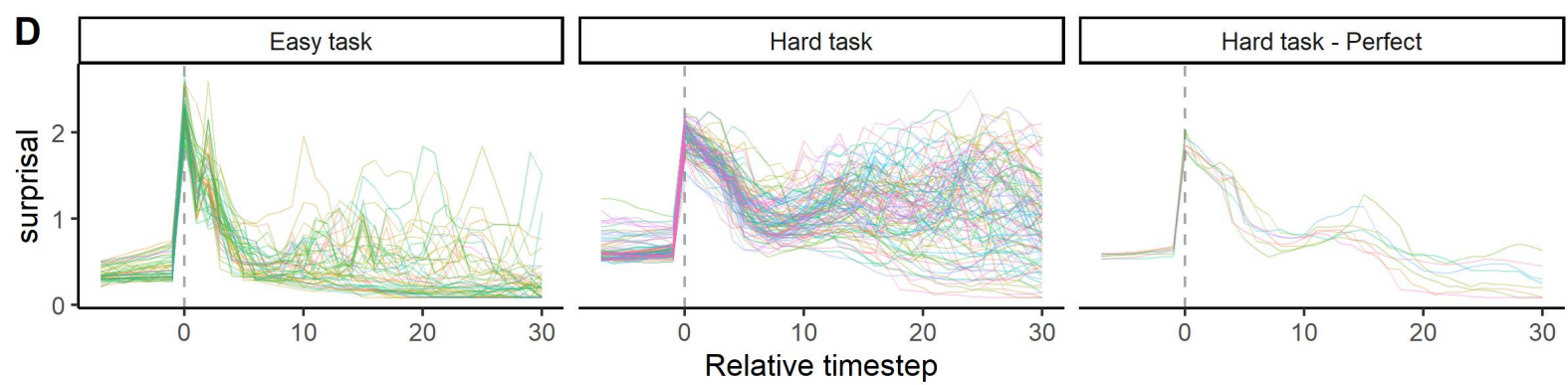

Supplement: S5 Fig — Trial-time fluctuations shown with each line of descent displayed separately. (PDF) [file pcbi.1011346.s005.pdf]

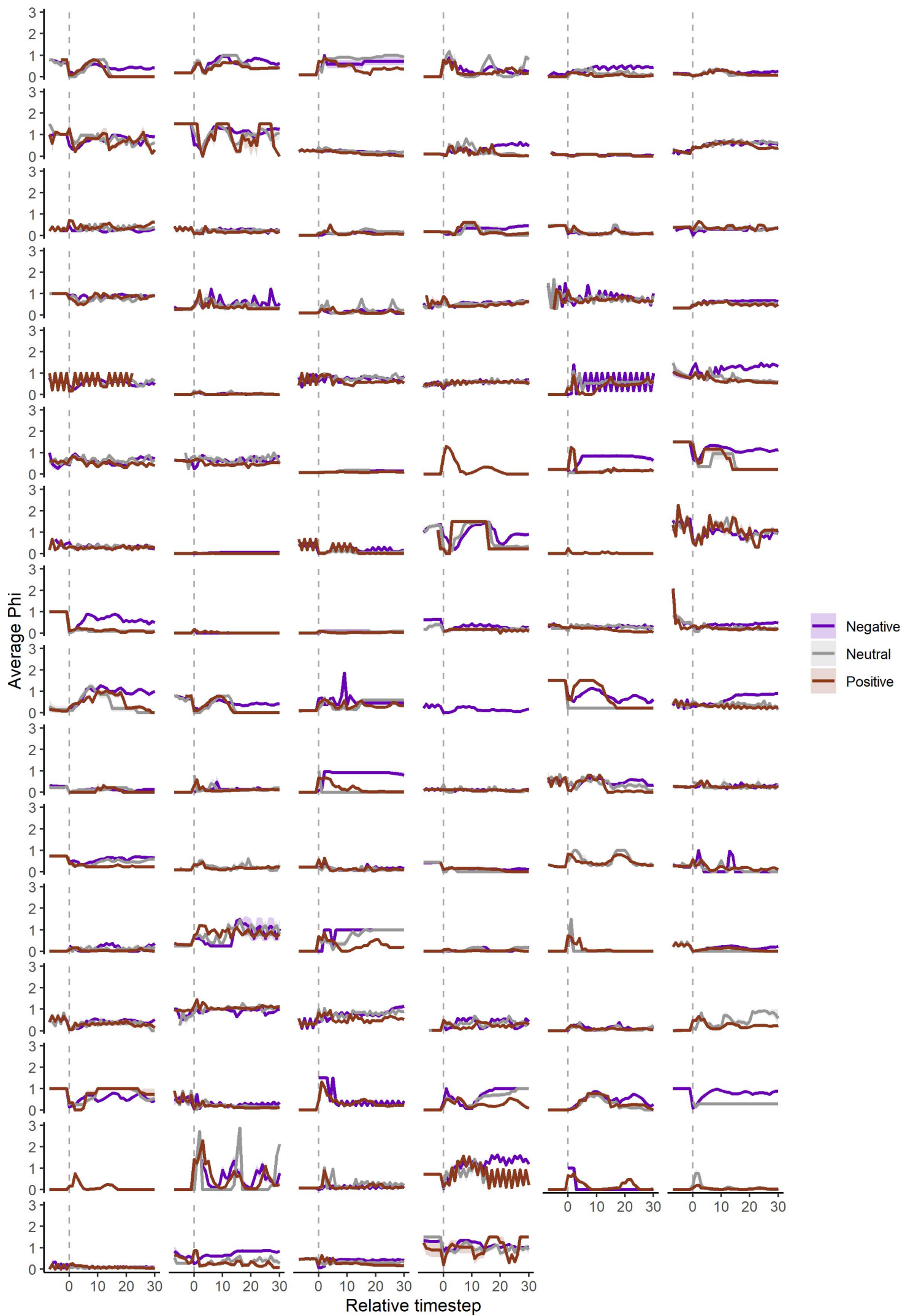

Supplement: S6 Fig — Trial-time fluctuations shown with each line of descent displayed separately. (PDF) [file pcbi.1011346.s006.pdf]

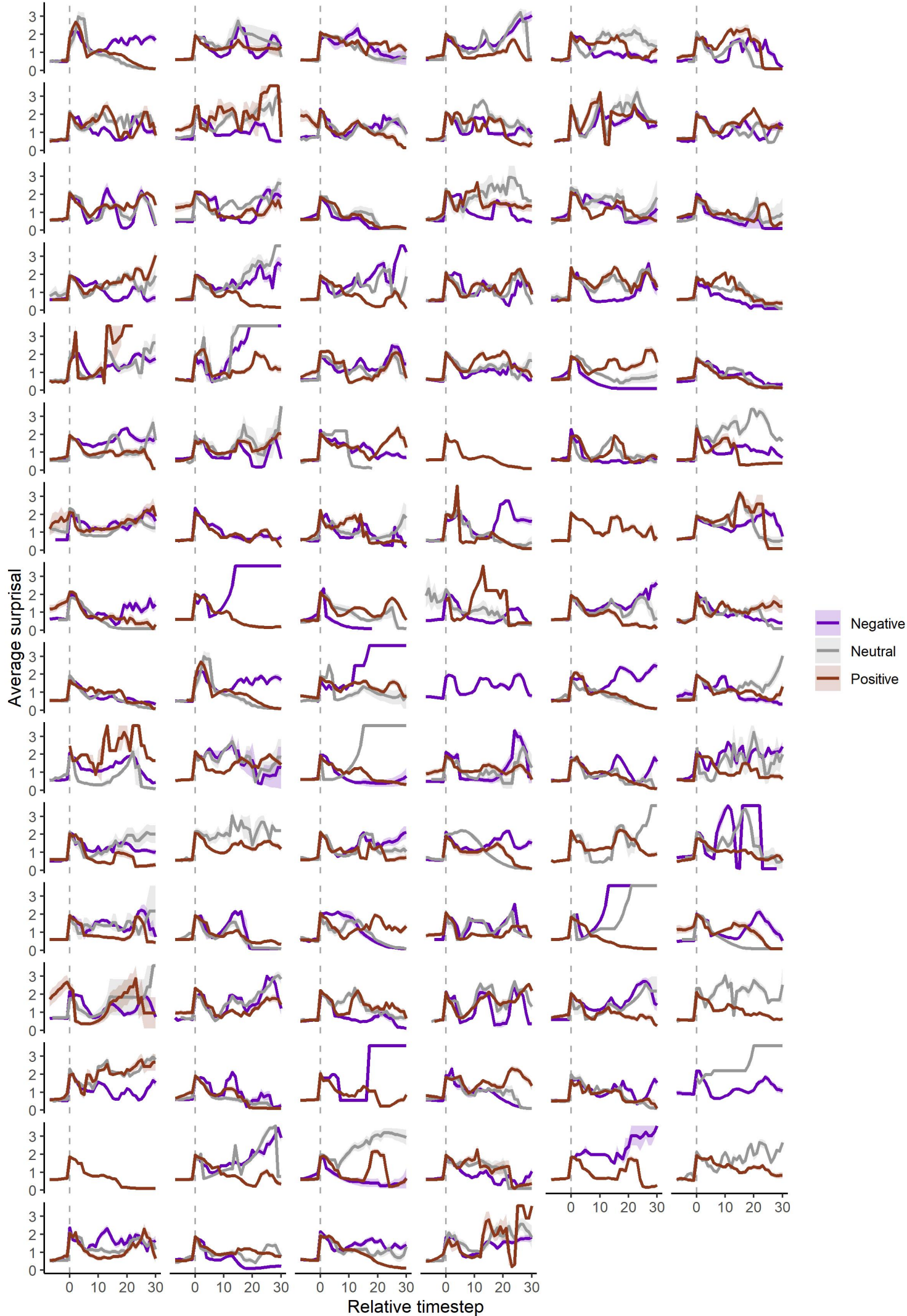

Supplement: S7 Fig — Trial-time fluctuations shown with each line of descent displayed separately. (PDF) [file pcbi.1011346.s007.pdf]
